# Intersectional stereotypes: How age and race shape perceptions of women in the United States

**DOI:** 10.1371/journal.pone.0361210

**Published:** 2026-10-26

**Authors:** Hannah Gans, Veronica N. Z. Bergstrom, Drishti Thakkar, Alison L. Chasteen

**Affiliations:** Department of Psychology, University of Toronto, Toronto, Ontario, Canada; Utah State University, UNITED STATES OF AMERICA

## Abstract

This study examined the influence of older age and race (White/Black/Arab) on stereotypes of women. To evaluate whether age and race shape stereotype content for an intersectional group relative to its superordinate racial or superordinate older age identities, 525 Amazon Mechanical Turk participants were asked to list up to five words or short phrases that most people associated with these target groups. Overall, the study found stronger support for a distinctiveness hypothesis, whereby the most distinct identity of an individual’s intersectional identity influenced their associated characteristics. For example, because being White is common in the United States, old White women had more stereotypes in common with their old superordinate identity than their White identity. This suggests that the nation’s population characteristics may be predictive of the stereotypes associated with different intersectional groups. Overall, this research reveals the complex interplay between stereotypes of age and race for women, underscoring the need to acknowledge intersectional identities and cultural norms. Such insights can enhance our pursuit of inclusive and accurate societal perceptions.

## Introduction

The American population has changed dramatically in the past several decades, with almost 40% of Americans identifying as a racial minority [1]. Despite these demographic shifts, discrimination against racial minorities remains pervasive [2]. In particular, Black and Arab people, whose populations are increasing, continue to experience high levels of institutional discrimination in contexts such as airport security, employment, and law enforcement [3,4]. In order to reduce prejudice and discrimination toward Black and Arab communities, it is important to gain a better understanding of the stereotypes that lead to such biased treatment.

In parallel with broader race-based demographic shifts, the proportion of older adults from racial minority groups in the United States is steadily rising. Older adults now make up a larger share of the population than ever before, with notable gender differences, such that older women from racial minority groups significantly outnumber older men from racial minority groups [1,5]. By 2060, the population aged 65 and older is expected to double, comprising nearly one-quarter of the U.S. population, with the racial and ethnic minority share of this age group projected to increase by 115%.

Given the growing population of racially diverse women over the age of 65 there is clearly a need to examine how age and racial identities interact for women. Although some research has begun to examine how race intersects with certain individual social identities, relatively few studies have investigated how multiple intersecting identities shape perceptions of racially diverse targets, a framework known as intersectionality [6,7]. Importantly, intersectionality is not only concerned with how identities combine at the individual level, but also with how broader systems of power and inequality, such as racism, sexism and ageism, interact to shape social experiences and patterns of disadvantages [8,9]. From this perspective, stereotypes are not just beliefs about demographic groups; they also reflect wider social hierarchies and long-standing systems of inequality and oppression that shape how different groups are viewed and treated. Attending to intersectionality is therefore especially critical in stereotype research, given its strong influence on person perception [10–13].

### White, Black, Arab women stereotypes

While White, Black, and Arab women all fall into the same superordinate category of “woman”, stereotypes associated with women from each racial group differ significantly. A useful framework for understanding these differences is the Stereotype Content Model (SCM), which proposes that group stereotypes are organized along two primary dimensions: warmth and competence [14]. Research using the SCM has shown that gender, race, and age categories are each associated with distinct patterns of warmth and competence stereotypes, suggesting that intersecting identities may produce complex and potentially conflicting stereotype profiles.

White women, for instance, have traditionally been positioned as the prototypical standard of femininity in Western contexts [10]. More specifically, White women are often stereotyped as attractive, friendly, emotionally warm, and socially desirable; characteristics that align with traditional feminine norms and correspond to relatively favorable perceptions on the warmth dimension of the SCM [12,15]. In contrast, stereotypes of Black women portray a very different picture, reflecting a highly racialized construction of femininity. Black women are frequently depicted as being unfeminine, angry, hateful, irrational, undesirable, bitter, and mean, characteristics commonly captured in the “Angry Black Woman” stereotype [16]. These portrayals tend to position Black women as lower in perceived warmth and, in some contexts, higher in perceived dominance relative to White women, reflecting racialized expectations related to threat and aggression. Arab women are stereotyped in yet another qualitatively distinct manner. Rather than being framed as aggressive or unfeminine, Arab women are often stereotyped as being illiterate, weak, dependent, oppressed, and submissive [10,17]. From an SCM perspective, these stereotypes may reflect paternalistic forms of prejudice in which groups are perceived as relatively warm but low in competence. Together, these patterns illustrate how race shapes divergent expectations of femininity even within the same gender category.

Importantly, these stereotypes do not emerge in isolation but are embedded within broader social and institutional systems that reinforce unequal treatment across domains such as employment, healthcare, media representation, and surveillance [8]. From an intersectional perspective, stereotypes therefore function not only as cognitive associations, but also as mechanisms through which social inequalities are reproduced and maintained [9].

Examining White, Black, and Arab women in the present study was theoretically motivated by the need to situate intersectional stereotypes within a clear comparative framework. More specifically, since White individuals are often treated, implicitly or explicitly, as the normative reference group in psychological research, including White women therefore provides a comparison against which the stereotyping of racially minoritized women can be interpreted [10]. In contrast, Black and Arab women were chosen because both groups experience high levels of institutional discrimination, yet are stereotyped along fundamentally different dimensions.

Black women are often stereotyped through themes of threat, hostility, and unfemininity, whereas and Arab women are more commonly characterized through stereotypes of passivity, oppression, and cultural otherness. These differences are theoretically important because they show that racialized femininity is not stereotyped in one consistent way, but instead, that different groups are associated with different kinds of biased assumptions.

For Black women, these stereotypes are deeply rooted in historically constructed controlling images such as the “Mammy,” the “Jezebel,” and the “Sapphire”, which emerged through slavery, colonialism, and systemic racism [18,19]. These qualitative patterns are consistent with intersectional analyses showing that stereotypes of Black women are shaped by historically embedded and socially shared representations that reflect overlapping racial and gendered expectations. Importantly, these are not merely historical artifacts but continue to structure contemporary perceptions across domains like healthcare, education, media, and employment, shaping how Black women are evaluated in both interpersonal and institutional contexts [20,21]. These stereotypes are therefore intertwined with structural racism and sexism, influencing not only interpersonal impressions but also access to resources, credibility, safety, and social power.

Arab women, by contrast, are often constructed through intersecting Orientalist and Islamophobic frames that position them as exoticized, oppressed, or threatening [22,23]. These representations are tied to broader geopolitical and institutional processes, including racialization, immigration discourse, religious discrimination, and post 9/11 surveillance practices, which shape how Arab women are perceived and treated within Western societies. Moreover, these representations operate at the intersection of race, ethnicity, religion, and geopolitics, producing a distinct stereotype structure that differs qualitatively from that applied to Black women or to White women. Despite the social significance of these dynamics, Arab women remain largely absent from psychological studies of stereotypes, particularly those examining intersections of age and gender, highlighting a critical gap that this study seeks to address.

As such, studying older White women alongside older Black and Arab women allows us to determine whether particular stereotypes are specific to race and ethnicity or reflect more general patterns related to age and gender. Although other racial and ethnic groups (e.g., Latina or Asian women) are also subject to important and understudied stereotypes, including all groups within a single study would limit theoretical precision and was not feasible.

Each group plays a distinct inferential role within this design. White women provide a comparison group, allowing stereotype content to be evaluated in relation to age and race without strong racial distinctiveness. Black women represent a context where race is highly salient and historically central, allowing examination of how racial distinctiveness shapes the stereotypes associated with the group. Arab women represent a different form of racial categorization in which cultural, ethnic, and religious cues are particularly salient, allowing assessment of how this type of distinctiveness influences stereotype content.

Although research on stereotype content has increasingly recognized the role of intersecting identities, much of this work has focused on male targets, particularly in studies exploring the interaction of race and age [24]. For instance, in a facial emotion change-detection study, participants perceived anger as appearing earlier and lasting longer on young compared to older Black male faces, suggesting that age-based stereotypes may offer a protective buffer against the “Black hostility” stereotype in older Black men. Whether similar processes occur for women, and whether they vary across racial groups with distinct stereotype profiles, remains largely unknown.

Likewise, far less is known about how older women from racially diverse backgrounds are perceived, despite the fact that both age and gender are highly salient social categories and that older women are often subject to unique, compounding stereotypes tied to both. Meta-analytic findings indicate that women are stereotyped as less warm as they age, a pattern not seen to the same extent for men [25]. Moreover, aging women are not expected to decrease their agentic behavior as much as aging men, suggesting that gendered expectations persist across the lifespan in distinct ways [26]. What remains unclear, however, is how these patterns shift when race is introduced as a third, intersecting dimension of identity.

Therefore, given the growing older adult population, and the increasing racial diversity of older women in particular, there is a need to **systematically examine how intersecting identities of age, gender, and race/ethnicity shape social perceptions**. Investigating these dynamics among **older White, Black, and Arab women represents a theoretically grounded approach to** understanding stereotype content at the intersection of multiple identities and helps move beyond the narrow and incomplete picture offered by previous research.

### The present study

The purpose of this line of work was to identify how stereotypes of White, Black, and Arab women change when taking into account their age. We examined this by assessing the amount of overlap between their intersectional and superordinate identities and then by assessing whether stereotype content shifted between the intersectional group and their superordinate racial or superordinate older age identities. In the absence of research examining how old age and race affect stereotype content for women, much of this research was exploratory in nature. Rather than treating the analyses as hypothesis-confirming, we explicitly framed the study as a comparison of competing explanatory accounts of how intersectional stereotypes are structured.

Our main objective was to test the intersectionality hypothesis. Intersectionality theory conceptualizes social categories as jointly shaped by systems of power such as racism, sexism, and ageism, which produce unique configurations of disadvantage that are not simply the result of adding the individual stereotypes together [10,24,27–30]. Consistent with intersectionality theory, we viewed intersectionality as more than just the combination of multiple identities. Instead, these identities are shaped by broader systems of power and inequality, such as racism, sexism, and ageism. As a result, stereotypes of intersectional groups reflect the distinct social meanings attached to these overlapping positions within society, rather than a simple blend of individual identity stereotypes. Although we expect that distinct stereotypes will emerge when considering the intersectional identities in our research [11,12,24,26,29,31,32], we do not have a priori hypotheses about which stereotypes an old age identity will offset for each group or which unique stereotypes may emerge.

In addition to an intersectionality hypothesis, other competing hypotheses may be drawn about the relative influence of an age identity on the stereotype content of racial groups. One possibility is that one of the two superordinate identities (i.e., race or age) would drive the generation of traits associated with an intersectional identity. For instance, the superordinate racial identity might drive stereotype content, such that there will be greater perceived overlap in listed traits between the intersectional identity and the traits listed for the superordinate racial identity. Traits listed for an older White woman, for example, may have more stereotype content overlap with the category ‘White’ compared to ‘old’. Alternatively, if the superordinate age identity influences the listing of traits for an intersectional identity, then there would be greater overlap between traits listed for a target’s intersectional identity and the traits listed for their superordinate old age identity. In this case, an older White woman may have more stereotype content overlap with the superordinate category of ‘old’ compared to ‘White’. Another possibility, in line with a distinctiveness effect [33], is that the most unique or uncommon aspects of an individual’s overlapping social identities (such as race, gender, or age) may have a stronger influence on the stereotypes they face. This idea comes from research in cognitive psychology, particularly Tversky’s diagnosticity principle [34], which suggests that a trait is most informative, or “diagnostic”, when it sets one person apart from others in a relevant situation. According to data from the United States Census Bureau’s [1] data, approximately 58% of the population identifies as White, 17% as older in age, and 12% as Black. Although the Census Bureau does not provide data on the Arab population, estimates from the Arab American Institute suggest they constitute less than 1% of the population. Therefore, as predicted by the distinctiveness hypothesis, older Arab and White women, whose racial identities are more distinctive than their age in the United States [35], are expected to share more stereotypes with their racial group than with their age group. Moreover, because being Black and old, respectively, are both comparably rare identities in the United States, it would be expected that Black women and old women would have the same amount of stereotype content in common with old Black women. This would align with what is predicted by the Lens-Based Model, which suggests that perceivers rely on socially meaningful category cues to make inferences about others, particularly when certain identities are viewed as especially diagnostic or salient within a given social context [36].

We assessed how stereotype content for older White, Black, and Arab women differ from their superordinate older adult category and from their superordinate racial group identity. Because “Black” refers to a race rather than a geographical location, we chose “Arab” instead of “Middle Eastern” to maintain a consistent group label for the stereotypes to function within. Moreover, to avoid socially desirable responding due to people’s motivations to come across as unbiased,^32^ we asked participants to report on perceptions of societal stereotypes, not their own beliefs.^7-8,33^ Therefore, participants reported up to five stereotypes that they believed society held about the following groups: White women, Black women, Arab women, old women, old White women, old Black women, and old Arab women.

Overall, the study design was intended to compare three broad explanatory frameworks: the intersectionality hypothesis; superordinate identity dominance (race vs. age); and a distinctiveness-based account, rather than to test a single directional set of predictions.

## Method

Ethics approval from the Social Sciences, Humanities & Education Research Ethics Board at the University of Toronto Protocol #37031 was obtained for this study.

### Participants

Five-hundred and twenty-five Amazon Mechanical Turk (MTurk) participants were compensated $1.00 USD for their participation in this study from 29/04/2019–30/04/2019. Based on Bergstrom et al. [37] who used a similar study design, we aimed to collect data from at least 80 participants per target group. Each participant was tasked with generating a list of up to five short words or phrases for four distinct target groups. Note that we also explored an additional identity of sexuality. However, for conciseness, those results are found in Supporting Information (data in S2 Text). The total target sample size was thus 400. The only inclusion criterion was that participants must reside in the United States. We checked IP addresses and geographic coordinates to ensure participants met this inclusion criteria. Duplicate IP addresses and worker IDs, participants who did not pass our attention item, and responses that were highly likely reported by bots (i.e., nonsensical multi-sentence responses) were excluded from analysis. The final sample size was 426 (82–89 participants provided stereotypes for each target group). The sample had a mean age of 37.10 (*SD* = 12.26) and 52.58% were male. The majority (75.35%) of participants were White, 8.92% were Black, and the remaining 15.73% identified with another racial group (see S1 Table for full demographic breakdown).

### Procedure

Each participant was asked to list up to five words or short phrases that most people associate with four of the following social groups: women, White women, Black women, Arab women, old women, old White women, old Black women, and old Arab women. Groups were assigned to participants such that they never reported traits for any superordinate identity more than once. For example, a participant would never be assigned to list traits associated with an identity that included Black and then another intersectional identity that included White or Arab. In order to balance the number of identities that each participant group listed traits for (i.e., four identities), we assigned the superordinate category ‘women’ to one of the participant groups. Furthermore, the presentation order of groups within each subset were randomized (see S1 Text for the five subsets of four groups that participants were assigned to). A series of demographic questions followed the reporting of traits.

### Demographics

Participants reported their age, gender, political orientation, sexual orientation, race, religion, and highest level of education (see S1 Table to view participants’ full demographic breakdown).

### Data analysis

The coding was carried out in accordance with the method described in previously conducted work.^36^ To start, the data was first cleaned by two research assistants (RAs). Phase 1 consisted of converting plural words to singular, as well as correcting typos. The second phase consisted of summing phrases into single words and truncating them to form meaningful roots including all terms with similar meanings (e.g., “terrorism” to “terror*”). As part of phase 3, a stereotype dictionary was created for each superordinate and intersectional group based on the frequency of each stereotype that was generated. RAs discussed any disagreements during any phase. The graduate supervisor was to be consulted in cases of no resolution; however, no cases of no resolution were encountered.

Next, synonyms from the stereotype dictionaries were combined under a single term (e.g., “beautiful” became “attractive”). We used an online thesaurus (http://ww.thesaurus.com) to decide how to group words together. To make these changes, spreadsheets were loaded into Google’s Open Refine Program, which enabled all changes to be made across groups simultaneously in order to maintain consistency. Lastly, words that had a single count and were not listed for any other groups were dropped from analyses.

Statistical testing was conducted in R Studio (*Version 1.3.959*). Firstly, the percentage of overlap between the intersectional and superordinate categories was calculated. Secondly, Fisher’s exact tests were performed on the top 10 stereotypes of all superordinate and intersectional groups to assess whether stereotype content shifted between the intersectional group and their superordinate racial, superordinate older age identity. When there was a tie for the 10^th^ top stereotype, we included all of those traits in analyses. This resulted in 26 tests for Arab women age comparisons, 22 tests for Black women age comparisons, and 22 tests for White women age comparisons. Consequently, we Bonferroni-adjusted our alpha levels for the omnibus tests to 0.05/26 = .0019, 0.05/22 = .0023, 0.05/22 = .0023, respectively. When these omnibus tests were significant, we computed additional pairwise testing with a Holm adjustment to pinpoint where the differences in stereotype content emerged between groups. Data and R script are available on the Open Science Framework: [35]

## Results

Below, we first report the analyses for the stereotype content overlap between each intersectional group and their superordinate racial and age identities. Following this, we report the top six stereotypes that participants listed per group.

### Stereotype content overlap

#### Race and age.

Comparing stereotype content overlap of each intersectional group with their superordinate racial and age identities, old White women shared more stereotype content with old women than White women. In contrast, old Black women had a similar level of overlap with Black women and old women. Lastly, old Arab women had more stereotype content overlap with Arab women than old women. (see Table 1 for specific percentage overlap).

**Table 1:** Stereotype content overlap with the racial women category.

Subgroup	Superordinate group	Overlap (%)
Old White women	White women	41.41
Old White women	Old women	69.39
Old Black women	Black women	56.03
Old Black women	Old women	54.08
Old Arab women	Arab women	62.86
Old Arab women	Old women	55.10

Comparing stereotype content overlap with the superordinate old age category, old White women had the greatest overlap with the old women category, followed by both old Black women and old Arab women.

### Top stereotypes by age

In the following section, we report the top five stereotypes per group. Fisher’s exact tests were performed on the top 10 stereotypes of all superordinate and intersectional groups to assess whether stereotype content shifted between the intersectional group and their superordinate racial or superordinate older age identities.

#### White women.

The top five stereotypes for White women were privileged, financially secure, arrogant, kind, and intelligent; the top five stereotypes for old women were kind, slow, cranky, frail, and weak; the top five stereotypes for old White women were kind, cranky, slow, intolerant, frail, and mean.

Among the 22 tested traits, old White women were reported to be less arrogant, financially secure, privileged, and selfish than White women and no differently than old women. They were also more likely to be labelled as a cook, cranky, frail, slow, weak, and wise than White women and no differently than old women. In contrast, old White women were reported to be less worn out than old women and no differently than White women. Lastly, old White women were labelled a cheap more often than either of their superordinate groups. The remaining 10 traits were reported a similar number of times for old White women as their superordinate identities (see Table 2 for Fisher’s Exact tests).

**Table 2:** Fisher’s exact results for White age stereotype content comparisons.

Word	White women	Old women	Old White women	Fisher’s exact test p-value
**Arrogant**	18_a_	1_b_	4_b_	< .001
**Attractive**	11	1	3	.003
**Bad driver**	1	12	10	.003
**Cheap**	0_a_	1_a_	9_b_	< .001
**Cook**	0_a_	11_b_	9_b_	< .001
**Cranky**	1_a_	17_b_	21_b_	< .001
**Entitled**	9	0	5	.004
**Financially secure**	21_a_	2_b_	8_b_	< .001
**Forgetful**	0	9	5	.004
**Frail**	0_a_	15_b_	12_b_	< .001
**Intelligent**	14	4	3	.004
**Intolerant**	8	3	13	.035
**Kind**	15	23	32	.026
**Mean**	7	5	12	.237
**Privileged**	25_a_	0_b_	0_b_	< .001
**Rude**	4	3	9	.224
**Selfish**	9_a_	4_a,b_	0_b_	.002
**Slow**	0_a_	19_b_	14_b_	< .001
**Stupid**	10	5	5	.263
**Weak**	1_a_	13_b_	9_b_	.002
**Wise**	0_a_	12_b_	10_b_	< .001
**Worn out**	0_a_	8_b_	0_a_	< .001

Note. Within each row, subscripts that differ are significantly different from each other (Bonferroni corrected adjustments of 0.05/22 = p 0.023)

In sum, old White women were more similar to White women than old women for one trait. In contrast, old White women were more similar to old women than White women for 10 traits. Lastly, old White women had one unique trait ascribed to their group.

#### Black women.

The top six stereotypes for Black women were loud, rude, poor, angry, stupid, and tough; the top six stereotypes for old women were kind, slow, cranky, frail, and weak; the top six stereotypes for old Black women were loud, wise, kind, religious, and rude.

Among the 22 tested traits, old Black women were reported to be less of a bad driver, forgetful, frail**,** slow, and weak than old women and no differently than Black women. They were also more likely to be labelled as big and poor than old women and no differently than Black women. In contrast, old Black women were reported to be less aggressive and rude than Black women and no differently than old women. They were also more likely to be labelled as a cook, cranky, kind, and wise than Black women and no differently than old women. For only a single trait (loud), were old Black women statistically different from both of their superordinate categories but were stereotyped as an average between the two groups. More specifically, old Black women were stereotyped as louder than old women but were stereotyped as less loud than Black women, thus depicting an averaging effect where they were stereotyped as falling in the middle of the superordinate groups. Lastly, old Black women were reported to be more religious than either of their superordinate groups. The remaining seven traits were reported a similar number of times for old Black women as their superordinate identities (see Table 3 for Fisher’s Exact tests).

**Table 3:** Fisher’s exact results for Black age stereotype content comparisons.

Word	Black women	Old women	Old Black women	Fisher’s exact test p-value
**Aggressive**	12_a_	0_b_	2_b_	< .001
**Angry**	15	5	11	.075
**Bad driver**	1_a_	12_b_	0_a_	< .001
**Big**	12_a_	0_b_	9_a_	< .001
**Cook**	1_a_	11_b_	11_b_	.002
**Cranky**	0_a_	17_b_	9_b_	< .001
**Degenerate**	12_a_	0_b_	4_a,b_	< .001
**Forgetful**	0_a_	9_b_	0_a_	< .001
**Frail**	0_a_	15_b_	2_a_	< .001
**Kind**	3_a_	23_b_	19_b_	< .001
**Loud**	44_a_	1_b_	22_c_	< .001
**Mean**	10	5	11	.284
**Poor**	16_a_	1_b_	10_a_	< .001
**Pushy**	10	0	6	.003
**Religious**	2_a_	3_a_	18_b_	< .001
**Rude**	25_a_	3_b_	12_b_	< .001
**Slow**	1_a_	19_b_	2_a_	< .001
**Stupid**	14	5	8	.122
**Tough**	14	2	10	.006
**Weak**	1_a_	13_b_	1_a_	< .001
**Wise**	0_a_	12_b_	21_b_	< .001
**Worn out**	0_a_	8_b_	2_a,b_	.002

Note. Within each row, subscripts that differ are significantly different from each other (Bonferroni corrected adjustments of 0.05/22 = p 0.023)

In sum, old Black women were labelled as more similar to Black women than old women for seven traits. In contrast, old Black women were labelled as more similar to old women than Black women for six traits. Old Black women were labelled as an average between their two superordinate groups for a single trait. Lastly, old Black women had one unique trait ascribed to their group.

#### Arab women.

The top six stereotypes for Arab women were oppressed, quiet, submissive, uneducated, religious, and wear hijab/veil; the top six stereotypes for old women were kind, slow, cranky, frail, and weak; the top six stereotypes for old Arab women were religious, old-fashioned, quiet, submissive, kind, mean, and discriminated against.

Among the 26 tested traits, old Arab women were reported to be less of a bad driver, forgetful, frail, slow, wise, and worn out than old women and no differently than Arab women. Old Arab women were also more likely to be labelled as a terrorist and wearing a hijab/veil than old women and no differently than Arab women. In contrast, old Arab women were reported to be less obedient and uneducated than Arab women and no differently than old women. For the following four traits, old Arab women were statistically different from both of their superordinate categories: cranky, oppressed, quiet, and submissive. However, for these traits they were consistently stereotyped as falling somewhere in between the two groups, implying an inclination towards an averaging effect in how they were depicted. For example, when it came to the trait of being “cranky,” old Arab women were found to be significantly different from both Arab women and old women in that they were seen as less cranky than old women but more cranky than Arab women. Lastly, old Arab women were reported to be more religious, discriminated against, and unjust than either of their superordinate groups. The remaining nine traits were reported a similar number of times for old Arab women as their superordinate identities (see Table 4 for Fisher’s Exact tests).

**Table 4:** Fisher’s exact results for Arab age stereotype content comparisons.

Word	Arab women	Old women	Old Arab women	Fisher’s exact test p-value
**Attractive**	8	1	3	.044
**Bad driver**	2_a_	12_b_	2_a_	.001
**Conservative**	3	5	10	.130
**Cook**	1	11	4	.005
**Cranky**	0_a_	17_b_	6_c_	< .001
**Discriminated against**	0_a_	0_a_	13_b_	< .001
**Forgetful**	0_a_	9_b_	1_a_	< .001
**Frail**	0_a_	15_b_	1_a_	< .001
**Intolerant**	4	3	12	.030
**Kind**	6_a_	23_b_	13_a,b_	.001
**Mean**	2	5	13	.008
**Obedient**	11_a_	0_b_	0_b_	< .001
**Old-fashioned**	4_a_	7_a_	19_b_	.001
**Oppressed**	37_a_	0_b_	6_c_	< .001
**Quiet**	36_a_	7_b_	17_c_	< .001
**Religious**	13_a_	3_b_	25_c_	< .001
**Slow**	0_a_	19_b_	0_a_	< .001
**Stupid**	8	5	8	.745
**Submissive**	28_a_	1_b_	13_c_	< .001
**Terror(ist)**	9_a_	0_b_	10_a_	.002
**Uneducated**	15_a_	1_b_	3_b_	< .001
**Unjust**	0_a_	0_a_	10_b_	< .001
**Weak**	11	13	5	.089
**Wear hijab/veil**	13_a_	0_b_	10_a_	< .001
**Wise**	0_a_	12_b_	1_a_	< .001
**Worn out**	0_a_	8_b_	0a	< .001

Note. Within each row, subscripts that differ are significantly different from each other (Bonferroni corrected adjustments of 0.05/26 = p 0.0019)

In sum, old Arab women were labelled as more similar to Arab women than old women for eight traits. In contrast, old Arab women were labelled as more similar to old women than Arab women for two traits. Old Arab women were labelled as an average between their two superordinate groups for four traits. Lastly, old Arab women had three unique traits ascribed to their group.

## Discussion

This study examined how stereotypes of White, Black, and Arab women change when age is taken into account. This study was exploratory in nature in that we were interested in testing whether stereotype content is best explained by an intersectionality hypothesis, which predicts that stereotypes of intersectional identities contain unique elements that are not simply the result of adding the individual stereotypes together [10,24,27–29], or by alternative explanations emphasizing the relative influence of race, age and perceived social distinctiveness. Importantly, the intersectionality framework adopted here conceptualizes identities not only as individual-level categories, but as socially embedded constructs shaped by broader systems of inequality, including racism, sexism, and ageism, which structure how groups are perceived and evaluated in context.

Although we expected distinct stereotypes to emerge for each intersectional group, we did not have a priori hypotheses about which specific stereotypes might emerge. In addition, we evaluated evidence for three competing hypotheses: 1) that superordinate racial identity would drive stereotype content, 2) that superordinate age identity would drive stereotype content, and 3) that the more socially distinctive superordinate identity would drive stereotype content.

Our findings provided partial support for the intersectionality hypothesis, as each intersectional group had at least one unique trait that was not present in either of their superordinate identities (race or age alone). This effect was especially pronounced among Arab women, for whom three unique traits emerged in the “old Arab women” category. However, it was also evident in the other groups, as one unique trait emerged for both old Black and old White women. These findings support intersectionality models of stereotyping,^6^ which propose that new stereotypes can arise from the combination of different social identities, rather than simply resulting from additive or averaged traits from each identity.

At the same time, our findings aligned more closely with the distinctiveness hypothesis [32], demonstrating that the influence of each superordinate identity on stereotype content was asymmetrical. Specifically, each intersectional group showed greater stereotype overlap with the superordinate identity that is more socially distinctive in the United States. Consistent with our definition of social distinctiveness as the extent to which an identity is rare and socially salient, old Arab women were more likely to be stereotyped in line with Arab women (a more distinctive group) than with old women. Old Black women, for whom both race and age are socially distinctive, showed a more balanced stereotype profile but ratings still leaned toward their racial identity. In contrast, old White women, whose racial identity is comparatively less socially distinctive, were primarily stereotyped in line with old age. Together, these findings suggest that perceived distinctiveness shapes which identity category exerts greater influence on stereotype content.

More broadly, these findings suggest that perceivers may rely on whichever identity dimension is most salient in a given context. This salience is not only cognitive, but instead, it is also shaped by social environments in which inequality determines which identities are most visible and emphasized. From this perspective, “distinctiveness” reflects not just how people perceive others, but also how social hierarchies make certain groups more noticeable than others. As a result, the influence of specific identities on stereotype content reflects both cognitive processes and the broader power structures that guide social attention. This interpretation aligns with lens-based models of stereotyping, which propose that perceivers apply context-dependent “lenses” that prioritize different identity dimensions depending on their social relevance, rather than combining identities in a simple additive way [13,36].

Although the distinctiveness effect has been primarily investigated in the context of memory recall and social categorization [32–34,37,38], this study demonstrates that these effects also extend to intersectional person perception. Rather than consistently generating new stereotypes for intersectional groups, perceivers may rely more heavily on whichever identity dimension is most socially salient within a given context.

In summary, we observed stronger evidence supporting a distinctiveness hypothesis than an intersectionality hypothesis across all three racial groups. These findings have important implications for understanding the experiences of older Black and Arab women, as it suggests that their experiences of discrimination may not be primarily shaped by “ageism plus sexism”, but instead reflect dynamic processes in which different forms of bias become activated depending on which identity is made most salient within specific structural and institutional contexts. This interpretation aligns with emerging theoretical work on age-centered racism, which argues that age and race do not operate as separate systems of disadvantage, but instead interact in ways that shape how racialized older adults are socially perceived, evaluated, and treated across contexts [39]. As a result, older Black and Arab women may encounter shifting forms of bias across domains such as healthcare, employment and everyday social interactions, where different aspects of identity become more or less salient depending on the setting. For example, older racialized women may be perceived through stereotypes tied to racial threat, foreignness, incompetence, dependency, or social exclusion depending on which identity dimension is foregrounded in a particular context. These dynamics may contribute to unequal treatment, reduced access to opportunities, and cumulative stressors that affect both mental and physical health over time.

These findings also have implications for intervention and policy. Broad anti-ageism initiatives that treat older adults as a uniform category may overlook how racialized and ethnically minoritized older women are perceived through different stereotype lenses. Instead, interventions may be more effective when they explicitly address how stereotypes vary across intersectional identities. For example, stereotype reduction campaigns should incorporate more diverse and culturally specific representations of aging, ensuring that older Black and Arab women are visible in ways that counter dominant, socially salient stereotypes. In healthcare and workplace settings, training programs may also benefit from explicitly stating how assumptions about competence, dependency, and social belonging can shift depending on which identity is most salient. Consistent with an age-centered racism framework, such interventions should also recognize that age-based biases themselves may become racialized, such that aging is interpreted differently depending on the racial or ethnic group being perceived [39]. At the structural level, these results underscore the importance of designing equity policies that do not treat racism, sexism, and ageism as separate systems, but instead recognize how they interact in shaping perception and treatment [40].

### Limitations

This study was a novel exploration into the dynamics of stereotypes among Arab, Black, and White women when taking age into account. However, it is important to acknowledge certain limitations. First, to minimize social desirability bias, we specifically asked participants to report stereotypes held by the majority rather than their personal beliefs. Therefore, the findings reflect participants’ awareness of prevailing stereotypes rather than necessarily their personal endorsements of them. Despite this, it is possible that participant race and participant sexual orientation could moderate our findings. However, due to a lack of diversity in our participant pool (with 75% of our sample being White), we were unable to assess the impact of participant race on stereotype content.

Second, the data were collected prior to 2020, and it is possible that some racial and gender stereotypes have shifted in the intervening years. Broader social and political events in recent years (ex., increased public attention to racial injustice, changing media representations, and shifts in public discourse around aging post COVID-19) may have influenced the expression or social acceptability of certain stereotypes today. As a result, the specific stereotype content observed in this study may not fully reflect current patterns of social perception, although the broader structural processes identified (e.g., the role of distinctiveness and salience) are likely more stable over time.

Third, in the questions regarding stereotypes about different age groups, no specific age range was provided, leaving us without knowledge of the age-related perceptions participants linked to these women. For example, some participants may have perceived the older age identities to comprise women aged over 50 years, whereas other participants may have perceived the older age identities to comprise women aged over 80 years.

Finally, because we did not measure the perceived distinctiveness of each identity, but rather based distinctiveness on the prevalence of each identity in the United States, we cannot determine whether the results reflect an awareness of group distinctiveness. Future research should seek to measure participants’ awareness of the prevalence of each identity in society.

### Future directions

Future research should strive to investigate how stereotypes differ among various racial groups and whether minority groups share similar stereotype beliefs towards other minority groups, as compared to majority group members. Additionally, given the diverse and multifaceted identities of Arab and Black individuals, it would be valuable for subsequent studies to examine whether ethnicity influences the content of stereotypes for these racial groups. Similarly, exploring how stereotype content shifts when individuals are prompted to consider characteristics associated with Arabs, Middle Easterners, or Muslims would contribute to a more comprehensive understanding.

## Conclusion

Because individuals belong to multiple social groups concurrently, it is imperative to foster a more comprehensive understanding of how diverse identities interact and influence the nature of stereotypes. Currently, there is a scarcity of studies that examine stereotypes associated with women concerning race and age. Therefore, this exploratory study serves as a fundamental contribution in the effort to understand shifts in stereotype content when considering overlooked intersectional identities. Consequently, this initial research can be leveraged to develop tailored interventions that work towards reducing discrimination faced by older White, Black, and Arab women.

## Supporting information

S1 Table Participant Demographics. (DOCX)

S1 Text Participant Conditions. (DOCX)

S2 Text Sexual Orientation Results. (DOCX)

## References

[1] United States CB. United States Census Bureau. Racial and ethnic diversity in the United States: 2010 census and 2020 census [Internet]. Available from: https://www.census.gov/library/visualizations/interactive/racial-and-ethnic-diversity-in-the-united-states-2010-and-2020-census.html

[2] Lee RT, Perez AD, Boykin CM, Mendoza-Denton R. On the prevalence of racial discrimination in the United States. PLoS One. 2019;14(1):e0210698. doi: 10.1371/journal.pone.0210698 30629706 PMC6328188

[3] Bleich SN, Findling MG, Casey LS, Blendon RJ, Benson JM, SteelFisher GK, et al. Discrimination in the United States: Experiences of black Americans. Health Serv Res. 2019;54 Suppl 2(Suppl 2):1399–408. doi: 10.1111/1475-6773.13220 31663124 PMC6864380

[4] Hummel D, Daassa M, Alshabani N, Felo L. Identifying as Muslim and American: The role of discrimination. Review of Religious Research. 2020;62(3):465–83. doi: 10.1007/s13644-020-00418-x

[5] Vespa J, Medina L, Armstrong DM. Population Estimates and Projections.

[6] Cole ER. Intersectionality and research in psychology. American Psychologist. 2009;64(3):170–80. 10.1037/a0014564 19348518

[7] Moradi B, Parent MC, Weis AS, Ouch S, Broad KL. Mapping the Travels of Intersectionality Scholarship: A Citation Network Analysis. Psychol Women Q. 2020;44(2):151–69. doi: 10.1177/0361684320902408 38736968 PMC11086954

[8] Agénor M. Future Directions for Incorporating Intersectionality Into Quantitative Population Health Research. Am J Public Health. 2020;110(6):803–6. doi: 10.2105/AJPH.2020.305610 32298180 PMC7204457

[9] Aguayo-Romero RA. (Re)centering Black Feminism Into Intersectionality Research. Am J Public Health. 2021;111(1):101–3. doi: 10.2105/AJPH.2020.306005 33326278 PMC7750622

[10] Ghavami N, Peplau LA. An intersectional analysis of gender and ethnic stereotypes. Psychology of Women Quarterly. 2012;37(1):113–27. doi: 10.1177/0361684312464203

[11] Hall EV, Hall AV, Galinsky AD, Phillips KW. MOSAIC: A Model of Stereotyping Through Associated and Intersectional Categories. AMR. 2019;44(3):643–72. doi: 10.5465/amr.2017.0109

[12] Freeman JB, Ambady N. A dynamic interactive theory of person construal. Psychol Rev. 2011;118(2):247–79. doi: 10.1037/a0022327 21355661

[13] Petsko CD, Bodenhausen GV. Multifarious person perception: How social perceivers manage the complexity of intersectional targets. Social & Personality Psych. 2019;14(2). doi: 10.1111/spc3.12518

[14] Cuddy AJC, Fiske ST, Glick P. Warmth and Competence as Universal Dimensions of Social Perception: The Stereotype Content Model and the BIAS Map. Adv Experimental Social Psychol. Elsevier. 2008. p. 61–149. doi: 10.1016/s0065-2601(07)00002-0

[15] Coltrane S, Messineo M. The perpetuation of subtle prejudice: race and gender imagery in 1990s television advertising. Sex Roles. 2000;42(5–6):363–89. doi: 10.1023/a:1007046204478

[16] Morgan M, Bennett D. Getting off of black women’s backs: Love Her or Leave Her Alone. Du Bois Rev. 2006;3(2):485–502. doi: 10.1017/s1742058x06060334

[17] Erentzen CA, Bergstrom VNZ, Zeng N, Chasteen AL. The gendered nature of Muslim and Christian stereotypes in the United States. Group Processes & Intergroup Relations. 2022. doi: 10.1177/136843022211380 PMC10665135 38021318

[18] Collins PH. Black Feminist Thought: Knowledge, Consciousness, and the Politics of Empowerment. Routledge. 2000. https://books.google.ca/books?id=cdtYsU3zR14C

[19] West C. Mammy, Jezebel, Sapphire, and their homegirls: Developing an “oppositional gaze” toward the images of Black women. 2008. p. 286–99.

[20] Crenshaw K. Mapping the margins: Intersectionality, identity politics, and violence against women of color. Stanford Law Review. 1991;43(6):1241. doi: 10.2307/1229039

[21] Rosette AS, Koval CZ, Ma A, Livingston R. Race matters for women leaders: Intersectional effects on agentic deficiencies and penalties. The Leadership Quarterly. 2016;27(3):429–45. doi: 10.1016/j.leaqua.2016.01.008

[22] Salari S. Invisible in aging research: Arab Americans, Middle Eastern immigrants, and Muslims in the United States. The Gerontologist. 2002;42(5):580–8. 10.1093/geront/42.5.580 12351793

[23] Shaheen JG. Reel Bad Arabs: How Hollywood Vilifies a People. The ANNALS of the American Academy of Political and Social Science. 2003;588(1):171–93.

[24] Kang SK, Chasteen AL. Beyond the double-jeopardy hypothesis: Assessing emotion on the faces of multiply-categorizable targets of prejudice. J Experimen Social Psychol. 2009;45(6):1281–5. doi: 10.1016/j.jesp.2009.07.002

[25] Kite ME, Stockdale GD, Whitley BE Jr, Johnson BT. Attitudes Toward Younger and Older Adults: An Updated Meta‐Analytic Review. Journal of Social Issues. 2005;61(2):241–66. doi: 10.1111/j.1540-4560.2005.00404.x

[26] Martin AE, North MS, Phillips KW. Intersectional escape: Older women elude agentic prescriptions more than older men. Pers Soc Psychol Bull. 2019;45(3):342–59. doi: 10.1177/0146167218784895 30084290

[27] Crenshaw K. Demarginalizing the intersection of race and sex: A black feminist critique of antidiscrimination doctrine, feminist theory and antiracist politics. University of Chicago Legal Forum. 1989;:139–67.

[28] Cole ER, Zucker AN. Black and White women’s perspectives on femininity. Cultur Divers Ethnic Minor Psychol. 2007;13(1):1–9. doi: 10.1037/1099-9809.13.1.1 17227171

[29] Kang SK, Chasteen AL, Cadieux J, Cary LA, Syeda M. Comparing young and older adults’ perceptions of conflicting stereotypes and multiply-categorizable individuals. Psychol Aging. 2014;29(3):469–81. doi: 10.1037/a0037551 25244468

[30] Rosenthal L, Overstreet NM, Khukhlovich A, Brown BE, Godfrey C, Albritton T. Content of, sources of, and responses to sexual stereotypes of Black and Latinx women and men in the United States: A qualitative intersectional exploration. Journal of Social Issues. 2020;76(4):921–48. doi: 10.1111/josi.12411

[31] Mair CA. Social ties and depression: An Intersectional Examination of Black and White community-dwelling older adults. J Appl Gerontol. 2009;29(6):667–96. doi: 10.1177/0733464809350167

[32] Neel R, Lassetter B. The stigma of perceived irrelevance: An affordance-management theory of interpersonal invisibility. Psychol Rev. 2019;126(5):634–59. doi: 10.1037/rev0000143 30688473

[33] Nelson LJ, Miller DT. The distinctiveness effect in social categorization: you are what makes you unusual. Psychol Sci. 1995;6(4):246–9.

[34] Tversky A. Features of similarity. Psychol Rev. 1977;84(4):327–52. doi: 10.1037/0033-295x.84.4.327

[35] AA demographics - NATIONAL-4.

[36] Petsko CD, Rosette AS, Bodenhausen GV. Through the looking glass: A lens-based account of intersectional stereotyping. J Pers Soc Psychol. 2022;123(4):763–87. doi: 10.1037/pspi0000382 35025602

[37] Bergstrom VNZ, Cadieux J, Thakkar D, Chasteen AL. Same view, different lens: How intersectional identities reduce Americans’ stereotypes of threat regarding Arab and Black men. Group Process Intergroup Relat. 2024;27(2):348–65. doi: 10.1177/13684302231153802 38344472 PMC10857900

[38] Fazio RH, Jackson JR, Dunton BC, Williams CJ. Variability in automatic activation as an unobtrusive measure of racial attitudes: a bona fide pipeline?. J Pers Soc Psychol. 1995;69(6):1013–27. doi: 10.1037//0022-3514.69.6.1013 8531054

[39] Smith-Tran A. Age-centered racism: An emergent framework for considering how ageism and racism intersect. Sociol Res Online. 2026;31(2).

[40] Hudson S, Myer A, Berney E. Stereotyping, prejudice, and discrimination at the intersection of race and gender: An intersectional theory primer. Social Personal Psychol Compass. 2024;18.

