# Supplementary material for: Intersectional stereotypes: How age and race shape perceptions of women in the United States
Source: PLoS One. 2026 Oct 26;21(10):e0361210. doi: 10.1371/journal.pone.0361210 (PMC13704870; doi:10.1371/journal.pone.0361210)
Supplement: Supplementary file 1 [file pone.0361210.s001.docx]

# **S1 Table. Participant Demographics**

| Characteristic | Percentage |
| --- | --- |
| **Gender** |  |
| Men | 52.58 |
| Women | 46.48 |
| Other/Prefer not to say | 0.94 |
| **Race** |  |
| White | 75.35 |
| Black | 8.92 |
| East/Southeast Asian | 5.16 |
| Latin, Central, and South American | 4.23 |
| Multiracial | 3.29 |
| South Asian | 2.35 |
| Aboriginal/Indigenous | 0.47 |
| Middle Eastern | 0.00 |
| Other | 0.23 |
| **Religion** |  |
| Christian | 47.65 |
| Agnostic | 18.31 |
| Atheist | 16.43 |
| None | 7.75 |
| Views from multiple religions | 2.58 |
| Islamic | 1.41 |
| Jewish | 1.41 |
| Buddhist | 1.17 |
| Hindu | 0.47 |
| Other | 2.82 |
| **Sexual Orientation** |  |
| Straight | 86.62 |
| Bisexual | 8.69 |
| Gay | 3.05 |
| Other/Prefer not to say | 1.64 |
| **Education** |  |
| Did not complete High School | 0.47 |
| High school graduate/GED | 31.22 |
| Bachelor’s degree, college diploma, or associate’s degree | 54.69 |
| Masters, Doctor, or Professional degree | 13.61 |
| **Political Orientation** |  |
| Liberal | 52.57 |
| Conservative | 31.47 |
| Neutral | 15.96 |
| **ESL** |  |
| English as first language | 97.42 |
