# Supplementary material for: Intersectional stereotypes: How age and race shape perceptions of women in the United States
Source: PLoS One. 2026 Oct 26;21(10):e0361210. doi: 10.1371/journal.pone.0361210 (PMC13704870; doi:10.1371/journal.pone.0361210)
Supplement: Supplementary file 2 [file pone.0361210.s002.docx]

**S2 Text. Participant Conditions**

Participants were randomly assigned to report traits for one of the following subsets of the 20 groups.

1. Young women, old Arab women, lesbian Black women, straight White women
2. Straight women, old Black women, young Arab women, lesbian White women
3. Women, straight Black women, old White women, Arab women
4. White women, lesbian Arab women, young Black women, old women
5. Young White women, Black women, straight Arab women, lesbian men

The order of the groups within each subset was randomized.
