# Supplementary material for: Intersectional stereotypes: How age and race shape perceptions of women in the United States
Source: PLoS One. 2026 Oct 26;21(10):e0361210. doi: 10.1371/journal.pone.0361210 (PMC13704870; doi:10.1371/journal.pone.0361210)
Supplement: Supplementary file 3 [file pone.0361210.s003.docx]

**S3 Text. Sexual Orientation Results**

**Stereotype Content Overlap**

**Race and Sexual Orientation**

Comparing stereotype content overlap of each intersectional group with their superordinate racial and sexual orientation identities, lesbian Arab women had similar stereotype content overlap with Arab women as lesbian women. In contrast, lesbian Black women had slightly more stereotype content overlap with lesbian women and Black women. Lastly, lesbian White women shared a great deal more stereotype content with lesbian women than White women (see Table 2 for specific percentage overlap).

Comparing stereotype content overlap with the superordinate lesbian category, lesbian White women had the greatest overlap with lesbian women, followed by both lesbian Black women and lesbian Arab women.

**Table 1. Stereotype Content Overlap with the Racial Women Category.**

| **Subgroup** | **Superordinate group** | **Overlap (%)** |
| --- | --- | --- |
| Lesbian Arab women | Arab women | 48.57 |
| Lesbian Arab women | Lesbian women | 51.58 |
| Lesbian Black women | Black women | 47.41 |
| Lesbian Black women | Lesbian women | 52.63 |
| Lesbian White women | White women | 39.06 |
| Lesbian White women | Lesbian women | 61.05 |

**Top Stereotypes**

**Sexual Orientation**

**Arab women.** The top five stereotypes for Arab women were oppressed, quiet, submissive, uneducated, religious, and wear hijab/veil; the top five stereotypes for lesbian women were masculine, intolerant, stereotypic fashion, aggressive, and ugly; the top five stereotypes for lesbian Arab women were sinful, closeted, filthy, rare, and rebellious.

Among the 31 tested traits, lesbian Arab women were labelled as less aggressive, athletic, intolerant, ugly, wrong, and wearing of stereotypic fashion than lesbian women and no differently than Arab women. In contrast, lesbian Arab women were labelled as less obedient, quiet, religious, submissive, uneducated, and wearing of a hijab/veil than Arab women and no differently than lesbian women. Additionally, they were labelled as homosexual more often than Arab women and no differently than lesbian women. For only a single trait (masculine), were lesbian Black women statistically different from both of their superordinate categories but were stereotyped as an average between the two groups. Lastly, lesbian Arab women were labelled as more ostracized, rare, rebellious, and sinful than either of their superordinate categories. The remaining 12 traits were reported a similar number of times for lesbian Arab women as their superordinate identities (see Table 6 for Fisher’s Exact tests).

In sum, lesbian Arab women were labelled more similar to Arab women than lesbian women for six traits. In contrast, lesbian Arab women were labelled more similar to lesbian women than Arab women for seven traits. Lesbian Arab women were labelled an average between their two superordinate groups for a single trait. Lastly, lesbian Arab women had four unique traits ascribed to their group.

**Table 2. Fisher’s Exact Results for Arab Sexual Orientation Stereotype Content Comparisons (p = .0016.**

| **Word** | **Arab women** | **Lesbian women** | **Lesbian Arab women** | **Fisher’s exact test p-value** |
| --- | --- | --- | --- | --- |
| **Aggressive** | 0_a_ | 14_b_ | 2_a_ | < .001 |
| **Angry** | 4 | 7 | 1 | .105 |
| **Athletic** | 0_a_ | 7_b_ | 0_a_ | .001 |
| **Attractive** | 8 | 7 | 3 | .312 |
| **Closeted** | 7 | 7 | 12 | .293 |
| **Confident** | 1 | 2 | 8 | .018 |
| **Degenerate** | 1 | 7 | 8 | .033 |
| **Filthy** | 7 | 10 | 12 | .426 |
| **Homosexual** | 0 | 9 | 9 | .002 |
| **Intolerant** | 4_a_ | 25_b_ | 5_a_ | < .001 |
| **Kind** | 6 | 7 | 8 | .812 |
| **Masculine** | 0_a_ | 81_b_ | 7_c_ | < .001 |
| **Mean** | 2 | 7 | 5 | .244 |
| **Obedient** | 11_a_ | 1_b_ | 0_b_ | < .001 |
| **Ostracized** | 1_a_ | 1_a_ | 10_b_ | < .001 |
| **Quiet** | 36_a_ | 0_b_ | 1_b_ | < .001 |
| **Rare** | 0_a_ | 1_b_ | 12_a_ | < .001 |
| **Rebellious** | 1_a_ | 1_a_ | 12_b_ | < .001 |
| **Religious** | 13_a_ | 0_b_ | 2_b_ | < .001 |
| **Sinful** | 0_a_ | 3_a_ | 14_b_ | < .001 |
| **Stereotypic fashion** | 2_a_ | 19_b_ | 0_a_ | < .001 |
| **Stupid** | 8 | 3 | 4 | .280 |
| **Submissive** | 28_a_ | 0_b_ | 0_b_ | < .001 |
| **Terrorist** | 9 | 0 | 7 | .003 |
| **Tough** | 3 | 12 | 4 | .027 |
| **Ugly** | 1_a_ | 14_b_ | 1_a_ | < .001 |
| **Uneducated** | 15_a_ | 0_b_ | 0_b_ | < .001 |
| **Weak** | 11 | 2 | 1 | .002 |
| **Wear hijab/veil** | 13_a_ | 0_b_ | 0_b_ | < .001 |
| **Weird** | 2 | 13 | 8 | .007 |
| **Wrong** | 0_a_ | 12_b_ | 3_a_ | < .001 |

**Black women.** The top five stereotypes for Black women were loud, rude, poor, angry, stupid, and tough; the top five stereotypes for lesbian women were masculine, intolerant, stereotypic fashion, aggressive, and ugly; the top five stereotypes for lesbian Black women were masculine, loud, stereotypic fashion, tough, and intolerant.

Among the 23 tested traits, lesbian Black women were never stereotyped more similarly to Black women than lesbian women. They were labelled as more homosexual and liberal than Black women and no differently than lesbian women. Additionally, they were labelled as less poor and rude than Black women and no differently than lesbian women. For three traits (intolerant, loud, masculine), Lesbian Black women were labelled as statistically different from both of their superordinate categories but were stereotyped as an average between the two groups. Lastly, lesbian Black women were more likely to be labelled as wearing stereotypic fashion than either of their superordinate groups. The remaining 15 traits were reported a similar number of times for lesbian Black women as their superordinate identities (see Table 7 for Fisher’s Exact tests).

In sum, lesbian Black women were not labelled as more similar to Black women than lesbian women for any traits. Rather, lesbian Black women were labelled more similar to lesbian than Black women for four traits. Lesbian Black women were labelled an average between their two superordinate groups for three traits. Lastly, lesbian Black women had one unique trait ascribed to their group.

**Table 3. Fisher’s Exact Results for Black Sexual Orientation Stereotype Content Comparisons (p = .0022)**

| **Word** | **Black women** | **Lesbian women** | **Lesbian Black women** | **Fisher’s exact test p-value** |
| --- | --- | --- | --- | --- |
| **Aggressive** | 12 | 14 | 12 | .927 |
| **Angry** | 15 | 7 | 9 | .182 |
| **Athletic** | 2 | 7 | 2 | .121 |
| **Attractive** | 7 | 7 | 0 | .010 |
| **Big** | 12 | 3 | 8 | .045 |
| **Closeted** | 0 | 3 | 7 | .020 |
| **Degenerate** | 12 | 7 | 6 | .278 |
| **Filthy** | 2 | 10 | 6 | .063 |
| **Homosexual** | 0_a_ | 9_b_ | 10_b_ | .001 |
| **Intolerant** | 2_a_ | 25_b_ | 13_c_ | < .001 |
| **Kind*** | 3 | 7 | 7 | .398 |
| **Liberal** | 0_a_ | 6_b_ | 12_b_ | < .001 |
| **Loud** | 44_a_ | 6_b_ | 23_c_ | < .001 |
| **Masculine** | 0_a_ | 81_b_ | 66_c_ | < .001 |
| **Mean** | 10 | 7 | 10 | .719 |
| **Poor** | 16_a_ | 0_b_ | 4_b_ | < .001 |
| **Pushy** | 10 | 2 | 3 | .025 |
| **Rude** | 25_a_ | 4_b_ | 12_b_ | < .001 |
| **Stereotypic fashion** | 3_a_ | 3_a_ | 19_b_ | < .001 |
| **Stupid** | 14 | 3 | 3 | .003 |
| **Tough** | 14 | 12 | 17 | .622 |
| **Ugly** | 3 | 14 | 10 | .018 |
| **Weird** | 0 | 8 | 4 | .008 |

**White women.** The top five stereotypes for White women were privileged, financially secure, arrogant, kind, and intelligent; the top five stereotypes for lesbian women were masculine, intolerant, stereotypic fashion, aggressive, and ugly; the top five stereotypes for lesbian White women were masculine, intolerant, tough, liberal, and womanly.

Among the 27 tested traits, lesbian White women were less likely to be labelled as wearing stereotypic fashion as lesbian women and no differently than White women. In contrast, they were more likely to be labelled as ugly than White women and no differently than lesbian women. They were also less likely to be labelled as entitled, financially secure, and privileged than White women and no differently than lesbian women. For a single trait (masculine), lesbian White women were labelled as statistically different from both of their superordinate categories but were stereotyped as an average between the two groups. Lastly, lesbian White women were more likely to be labelled as womanly compared with both of their superordinate groups. The remaining 20 traits were reported a similar number of times for lesbian White women as their superordinate identities (see Table 8 for Fisher’s Exact tests).

In sum, lesbian White women were labelled more similar to White women than lesbian women for one trait. In contrast, lesbian White women were labelled more similar to lesbian women than White women for four traits. Lesbian White women were labelled an average between their two superordinate groups for a single trait. Lastly, lesbian White women had one unique trait ascribed to their group.

**Table 4. Fisher’s Exact Results for White Stereotype Content Comparisons (p = .0019).**

| **Word** | **White women** | **Lesbian women** | **Lesbian White women** | **Fisher’s exact test p-value** |
| --- | --- | --- | --- | --- |
| **Aggressive** | 0_a_ | 14_b_ | 6_a,b_ | < .001 |
| **Angry** | 2 | 7 | 5 | .293 |
| **Arrogant** | 18_a_ | 2_b_ | 7_a.b_ | < .001 |
| **Athletic** | 1 | 7 | 9 | .027 |
| **Attractive** | 11 | 7 | 8 | .537 |
| **Closeted** | 0 | 7 | 4 | .022 |
| **Degenerate** | 1 | 7 | 7 | .063 |
| **Entitled** | 9_a_ | 0_b_ | 1_b_ | < .001 |
| **Filthy** | 0 | 10 | 4 | .002 |
| **Financially secure** | 21_a_ | 0 | 0 | < .001 |
| **Homosexual** | 0 | 9 | 4 | .005 |
| **Intelligent** | 14 | 3 | 5 | .007 |
| **Intolerant** | 8 | 25 | 20 | .005 |
| **Kind*** | 15 | 7 | 3 | .007 |
| **Liberal** | 1 | 6 | 12 | .004 |
| **Loud** | 2 | 6 | 8 | .164 |
| **Masculine** | 0_a_ | 81_b_ | 56_c_ | < .001 |
| **Mean** | 7 | 7 | 9 | .856 |
| **Privileged** | 25_a_ | 0_b_ | 0_b_ | < .001 |
| **Selfish** | 9_a_ | 0_b_ | 3_a,b_ | .002 |
| **Short hair** | 0_a_ | 0_a_ | 8_b_ | < .001 |
| **Stereotypic fashion** | 0_a_ | 19_b_ | 2_a_ | < .001 |
| **Stupid** | 10 | 3 | 2 | .023 |
| **Tough** | 5 | 12 | 18 | .013 |
| **Ugly** | 0_a_ | 14_b_ | 9_b_ | < .001 |
| **Weird** | 1 | 8 | 5 | .069 |
| **Womanly** | 0_a_ | 0_a_ | 10_b_ | < .001 |
