# Supplementary material for: Intersectional stereotypes: How age and race shape perceptions of women in the United States
Source: PLoS One. 2026 Oct 26;21(10):e0361210. doi: 10.1371/journal.pone.0361210 (PMC13704870; doi:10.1371/journal.pone.0361210)
Supplement: Supplementary file 6 [file pone.0361210.s007.docx]

07-July-2026

Dr. Emily Chenette

Editor in-Chief, *PLOS One*

Dear Dr. Chenette,

Thank you for the thoughtful review of the second revision our manuscript PONE-D-25-56215 entitled " Intersectional Stereotypes: How Age and Race Shape Perceptions of Women in the United States". We greatly appreciate that the reviewers noted that our revised manuscript continues to address an important topic, and that the paper has the potential to provide a meaningful contribution by providing insight into how race and age intersect with gender to impact person perception.

Below, we address the final points made by the reviewers. We consider this third revision of the paper, which we have revised in accordance with the suggestions provided, to have strengthened the manuscript. We hope that our revisions address your concerns and that you will now find the paper to be suitable for publication.

Best regards,

Hannah Gans, Alison L. Chasteen.

**Reviewer #4:** Thanks for the revisions and one concern remains. The following article which was mentioned in my previous round review and is relevant and should be reviewed and discussed in the current manuscript.
Rosenthal, L., Overstreet, N. M., Khukhlovich, A., Brown, B. E., Godfrey, C. J., &
Albritton, T. (2020). Content of, sources of, and responses to sexual stereotypes of
Black and Latinx women and men in the United States: A qualitative intersectional
exploration. Journal of Social Issues, 76(4), 921-948.

**Response:** Thank you for catching this oversight. We have reviewed this article and have integrated it into the manuscript on page 9 line 1.

**Reviewer #5:** Thank you for your revision. Most of my comments have been addressed, and the additional justification you have provided on page 8-9 regarding why Black and Arab female stereotypes was especially helpful.

However, in light of that, I do think that it would be worthwhile to remove the sentence on page 8, lines 5-7: "The present approach prioritizes depth over breadth by focusing on groups with well-documented but contrasting stereotype profiles." What I wrote in my original review was that this particular phrase essentially states that Asian and Latina women DO NOT have "well-documented but contrasting stereotype profiles."

To be clear, I think your justification on pages 8-9 is sufficient, and I do not disagree with you on sample feasibility. But not only do I find this statement to be empirically untrue, it risks reinforcing stereotypes about Asian/Latina women - especially hard to reconcile with the subject of this paper. Given your excellent explanation afterward, I'm unsure why it was left in.

**Response:** Thank you for this recommendation. We agree with this and have removed the sentence on page 8 lines 5-7.
