# Supplementary material for: Intersectional stereotypes: How age and race shape perceptions of women in the United States
Source: PLoS One. 2026 Oct 26;21(10):e0361210. doi: 10.1371/journal.pone.0361210 (PMC13704870; doi:10.1371/journal.pone.0361210)
Supplement: Supplementary file 7 [file pone.0361210.s005.docx]

12-Jan-2026

Dr. Emily Chenette

Editor in-Chief, *PLOS One*

Dear Dr. Chenette,

Thank you for the thoughtful review of our manuscript PONE-D-25-56215 entitled " Intersectional Stereotypes: How Age and Race Shape Perceptions of Women in the United States". We greatly appreciate that the reviewers noted that our manuscript addresses an important topic, and that the paper has the potential to provide a meaningful contribution by providing insight into how race and age intersect with gender to impact person perception.

Below, we address each point made by you and the reviewers. We consider the current version of the paper, which we have revised in accordance with the suggestions provided, to have strengthened the manuscript. We hope that our revisions address your concerns and that you will now find the paper to be suitable for publication.

Best regards,

Hannah Gans, Alison L. Chasteen.

**Reviewer #1’s comments:**

This manuscript examines how intersecting identities of age and race influence stereotypes about women, focusing on older women across three racial groups (Black, White, and Arab). The topic is timely and relevant, given the growing diversity and aging of the U.S. population. The study addresses an important gap by exploring stereotype content at the intersection of age and race for women – a novel contribution, since much past work has focused on either one dimension or on male targets. The manuscript’s strengths include its novelty (investigating understudied combinations of social identities), the thorough data collection and coding of stereotype descriptors from a large sample, and the effort to integrate theoretical frameworks (e.g., intersectionality and distinctiveness principles). These qualities suggest the work has potential to contribute meaningfully to the literature.

However, there are several issues that need to be addressed to strengthen the manuscript. I outline below the major concerns, followed by minor points and general recommendations for improving clarity and tone. My aim is to provide constructive feedback that will help the authors refine their work.

1) Title Wording (“Shift” Implications): The title “How intersectional identities shift stereotypes for women in the United States…” could be interpreted as implying a causal or dynamic change. The word “shift” suggests that something about having an intersectional identity causes stereotypes to change in a particular direction. However, the study design is cross-sectional and descriptive, comparing stereotype content across groups. There is no temporal shift measured, nor an experimental manipulation to show causation. To avoid misinterpretation, the authors should consider rewording the title to better reflect the content. For example, “How intersectional identities are associated with stereotypes…” or “Intersectional identities and stereotype content: The case of age and race for women in the U.S.”. Such formulations would accurately convey that the study examines differences or associations, rather than implying a causal shift. A clearer title will ensure readers understand the nature of the findings from the outset.

**Response:** Thank you for raising this suggestion. We have rewritten the title to better reflect the content.

2) Some statements in the Introduction come across as advocacy-driven or overly absolute, which may undermine the scholarly tone. For example, the manuscript states that “Black and Arab people… face some of the most discriminatory treatment at an institutional level…”. This is a strong, broad claim. Even if supported by citations, phrasing it as “the most discriminatory treatment” makes it sound absolute and potentially subjective. I recommend rephrasing such sentences to maintain an objective, evidence-based tone. For instance, the authors could say “Black and Arab people … face very high levels of institutional discrimination (in contexts such as airport security, employment, and law enforcement)”, and ensure each assertion is backed by a citation. This wording conveys the severity of discrimination without implying an unqualified superlative. Overall, the authors should aim to balance the introduction’s passionate framing with neutral scholarly language. This will help the paper come across as objective research rather than advocacy, which in turn can make its conclusions more credible to a scientific audience.
The statement, “To date, most research on people of color has focused on racial groups as a monolith…” overlooks a substantial body of work that has examined intersecting identities beyond race. For instance, numerous studies have explored how factors such as first-generation college student status, socioeconomic class, gender identity, and immigration status intersect with race to shape perceptions and experiences. The authors should revise this claim to more accurately reflect the existing literature and avoid overgeneralization. A more precise framing would strengthen the credibility and scholarly balance of the introduction.

**Response:** Thank you for this recommendation. We have refined several areas of the text to reflect more neutral scholarly language rather than advocacy driven statements (see pages 3-4).

3) Rationale for Selecting Specific Racial Groups: The choice to focus on Black, White, and Arab women needs a clearer rationale and more consistent presentation. It is valuable that the study goes beyond the Black/White binary; however, the inclusion of Arab women (and not other racial/ethnic groups such as Latina or Asian women) is not fully explained. The Introduction notes that Black and Arab people face high levels of discrimination, but the authors should explicitly clarify why these three groups were selected for analysis over other groups. Is it due to their unique demographic distinctiveness, particular social relevance, or previous literature on these groups? Providing a rationale will help readers understand the scope of the study. Additionally, the manuscript should maintain a consistent order when referring to these groups (sometimes the text reads “Black, White, Arab” and other times “Arab, Black, White”). Choosing one ordering (for example, alphabetical or by population size) and using it throughout will avoid confusion. Clarifying the group selection and ensuring consistency in references will strengthen the study’s focus and coherence. Additionally, if White women are used as a reference group to compare the marginalization of Black and Arab women, the authors should clearly explain why. A brief justification rooted in social hierarchy or prior literature would help strengthen the theoretical framework and avoid confusion.

**Response:** Thank you for these suggestions. Because of word count, we are unable to include as many details on the rationale for our choice as we would have liked. However, we have integrated as many relevant details as we could fit (see pages 4-6).

4) Inconsistent Ordering of Racial Categories: As noted, the manuscript alternates between listing racial groups in different sequences (“Black, White, Arab” in some places versus “Arab, Black, White” in others). This inconsistency can distract or confuse readers. It would be best to decide on one ordering convention and apply it uniformly throughout the paper. For instance, the authors might choose alphabetical order (Arab, Black, White) or another logical order, but they should stick with it for all mentions of the three groups.

**Response:** Thank you for raising these concerns. We have now ordered these groups consistently throughout.

5) Use of Discipline-Specific Jargon: The text includes quite a bit of specialized terminology (e.g., “intersectional stereotyping,” “diagnosticity principle,” “distinctiveness hypothesis,” “emergent trait content”). While these terms are familiar within social psychology, they may alienate or confuse readers from other backgrounds. To improve accessibility, the authors should define or simplify jargon where possible. For example, when first mentioning the “diagnosticity principle,” provide a brief explanation in plain language (the manuscript does this to some extent by noting it’s about a feature being informative when it distinguishes an individual, which is helpful). Similarly, phrases like “emergent trait content” could be unpacked (e.g., explain that it refers to new stereotype attributes that appear uniquely for the combined identity, rather than being a simple mix of the two single identities’ stereotypes). Ensuring that the prose is understandable to a broad audience will increase the impact of the work, especially in a general-interest journal.

**Response:** Thank you for this suggestion. We have explained the terms respectively throughout (see pages 3 and page 8).

6) Phrasing and Grammar: There are a few instances of awkward or unclear phrasing and minor grammatical issues. The authors should carefully proofread and possibly have someone not involved in the study read the manuscript to catch confusing sentences. For example, some sentences in the literature review are quite long or double up on ideas, making them hard to follow. Breaking long sentences into two and removing redundant phrases would help. Also, any minor typographical errors or formatting glitches (like inconsistent capitalization, extra spaces, or artifacts from references) should be fixed to polish the presentation. These edits will improve the overall readability and professionalism of the paper.

**Response:** Thank you for drawing our attention to this. We have done our best to edit the manuscript to make it easier to read.

**Reviewer #2’s comments**

This is a clear and focused study on stereotypes, examining whether stereotypes of older women vary by race (Arab, Black, White) and whether the “most distinctive” identity dimension dominates stereotype content. The design choice – using free-response measures rather than fixed scales – is a particular strength of the study, as it captures culturally available stereotype content without imposing researcher-defined categories.
After a careful review of the data and analyses, I recommend publishing this study. It represents a meaningful contribution, whose strongest claims are transparent, high-quality coding and careful interpretation.

1) I noted one minor error on page 13: the variable titles in Table 2 appear to be duplicated in the seventh line of the table, likely due to a page break.

**Response:** Thank you for catching this. We have fixed the table accordingly.
