# Supplementary material for: Intersectional stereotypes: How age and race shape perceptions of women in the United States
Source: PLoS One. 2026 Oct 26;21(10):e0361210. doi: 10.1371/journal.pone.0361210 (PMC13704870; doi:10.1371/journal.pone.0361210)
Supplement: Supplementary file 8 [file pone.0361210.s006.docx]

22-June-2026

Dr. Emily Chenette

Editor in-Chief, *PLOS One*

Dear Dr. Chenette,

Thank you for the thoughtful review of the first revision our manuscript PONE-D-25-56215 entitled " Intersectional Stereotypes: How Age and Race Shape Perceptions of Women in the United States". We greatly appreciate that the reviewers noted that our revised manuscript continues to address an important topic, and that the paper has the potential to provide a meaningful contribution by providing insight into how race and age intersect with gender to impact person perception.

Below, we address each point made by you and the reviewers. We consider this second revision of the paper, which we have revised in accordance with the suggestions provided, to have strengthened the manuscript. We hope that our revisions address your concerns and that you will now find the paper to be suitable for publication.

Best regards,

Hannah Gans, Alison L. Chasteen.

**Reviewer #3:** The authors should be commended for their thoughtful work moving the literature forward. The authors have clearly addressed prior reviewer comments. Below are suggestions to strengthen the manuscript, especially regarding intersectionality and presentation of findings.

We thank the reviewer for their positive comments regarding the potential contribution of this work and of our efforts at addressing reviewer suggestions.

**Major suggestion:**
1) Intersectionality: The manuscript currently treats intersectionality mainly as an analytic framework for intersecting individual identities. Expand this to emphasize that intersectionality links intersecting identities (e.g., race, gender, age) to social structures and processes (e.g., racism, sexism, ageism) that produce unequal outcomes. This theoretical expansion should be reflected in the definition, analytic approach, interpretation of results, and implications. Useful references to incorporate:
Agénor, M. (2020). Future directions for incorporating intersectionality into quantitative population health research. American Journal of Public Health, 110(6), 803–806.
Aguayo-Romero, R. A. (2021). (Re)centering Black feminism into intersectionality research. American Journal of Public Health, 111(1), 101–103.
Moradi, B., Parent, M. C., Weis, A. S., Ouch, S., & Broad, K. L. (2020). Mapping the travels of intersectionality scholarship: A citation network analysis. Psychology of Women Quarterly, 44(2), 151–169.

**Response:** Thank you for these suggestions. We have expanded the way intersectionality is described to reflect the fact that it links intersecting identities (e.g., race, gender, age) to social structures and processes (e.g., racism, sexism, ageism) that produce unequal outcomes, and have incorporated the recommended references in these areas (see page 3, lines 20-23- page 4, lines 1-3; page 9, lines 4-12; page 20, lines 10-13).

2)Interpretation: Where the results are discussed as supporting a “distinctiveness hypothesis” rather than an “intersectional hypothesis,” clarify what this means substantively. Explain implications for understanding how group-specific stereotypes or experiences operate, and how structural processes (rather than only individual identity combinations) might explain these patterns.

**Response:** Thank you for this recommendation. We have described the implications for our results in further detail (page 22, lines 17-23; page 23, lines 1-23).

**Specific edits / minor comments:**

1) Pg. 6, line 77: Expand the definition of intersectionality to state explicitly that it is not only about intersecting individual identities but about how those identities are linked to and produced by social structures and power relations (e.g., racism, sexism, classism).

**Response:** Thank you for this suggestion. We have expanded the way intersectionality is described to reflect the fact that it links intersecting identities (e.g., race, gender, age) to social structures and processes (e.g., racism, sexism, ageism) that produce unequal outcomes.

2) Pg. 17, line 321: Check count — there are six stereotypes listed for older White women, not five.
Pg. 20, line 370: There are six stereotypes listed for Black women — please verify and correct count/description. Pg. 22, line 400: There are six stereotypes listed for Arab women and older Arab women — please verify and correct.

**Response:** Thank you for catching this oversight. We have verified and corrected these numbers.

3) Pg. 27, line 488: Clarify the meaning and implications of finding more evidence for a distinctiveness hypothesis than an intersectional hypothesis. What does this imply about mechanisms, policy, or intervention priorities?

**Response:** Thank you for this suggestion. We have clarified the meaning and implications for finding mor evidence for a distinctiveness hypothesis compared to an intersectional hypothesis (page 22, lines 17-23; page 23, lines 1-23).

**Implications and recommendations:**

1) Expand the implications section. Specifically spell out what the findings mean for Black and Arab women in practical terms (e.g., experiences of discrimination, health, social opportunities).

**Response:** Thank you for this suggestion. We have expanded on what the findings mean for Black and Arab women in practical terms (see Page 23, Lines 1-7).

2) Discuss how these results could inform tailored interventions or policies (e.g., stereotype reduction campaigns, culturally specific support programs, structural interventions to address racism/sexism). If possible, suggest concrete next steps for research that would test structural mechanisms (e.g., measures of exposure to racism or sexism, policy variation, multilevel models).

**Response:** Thank you for this suggestion. We have expanded on how these results could inform tailored interventions or policies, as well as concrete next steps for research (see Page 23, Lines 8-23)

**Reviewer #4:** Overall assessment, this is a strong and important manuscript with careful revisions already completed. Strengthening the conceptualization and application of and expanding the implications and interpretation will substantially improve the paper’s contribution.

Thanks for the invitation. I am not the original reviewer of this manuscript. This investigation aimed to examine the influence of older age and race (White/Black/Arab) on stereotypes of women. The study found stronger support for a distinctiveness hypothesis, whereby the most distinct identity of an individual's intersectional identity influenced their associated characteristics. Overall, this topic is interesting, and the findings hold some theoretical and practical implications. However, some concerns appeared after reading the whole manuscript.

**Response:** We are gratified that the Reviewer feels this is a strong and important manuscript and that the topic is interesting and that the findings hold theoretical and practical implications. We also appreciate the acknowledgement that we had already completed careful revisions and the suggestions for further strengthening of the paper.

1) Some important papers need to be reviewed and discussed, such as,
Hudson, S. K. T. J., Myer, A., & Berney, E. C. (2024). Stereotyping, prejudice, and discrimination at the intersection of race and gender: An intersectional theory primer. Social and Personality Psychology Compass, 18(2), e12939.
Petsko, C. D., Rosette, A. S., & Bodenhausen, G. V. (2022). Through the looking glass: A lens-based account of intersectional stereotyping. Journal of personality and social psychology, 123(4), 763.
Rosenthal, L., Overstreet, N. M., Khukhlovich, A., Brown, B. E., Godfrey, C. J., & Albritton, T. (2020). Content of, sources of, and responses to sexual stereotypes of Black and Latinx women and men in the United States: A qualitative intersectional exploration. Journal of Social Issues, 76(4), 921-948.
Hand, M. D., & Ihara, E. S. (2024). Ageism, racism, sexism, and work with older healthcare clients: Why an intersectional approach is needed in practice, policy, education, and research. The International Journal of Aging and Human Development, 98(1), 27-38.

**Response:** Thank you for this suggestion. We have included these references throughout.

2) The theoretical implication can be further discussed with the follow framework.
Smith-Tran, A. (2025). Age-Centered Racism: An Emergent Framework for Considering How Ageism and Racism Intersect. Sociological Research Online, 13607804251382021.

**Response:** Thank you for this recommendation. We have expanded the theoretical implications with the use of these references (Page 22, lines 17-23; Page 23, lines 1-23).

3) For the stereotype content of White, Black, Arab Women Stereotypes, I believe the findings from the Stereotype Content Model would provide much more directly-related evidence and should be reviewed and discussed in the current manuscript. Please do not worry about the word limit since the Plos one is an open access journal without specific page limit.

**Response:** Thank you for this recommendation. We have included a description of the Stereotype Content Model as well as predictions based off of this model into the manuscript (see Page 4, Lines 9-14, Lines 17-19; Page 5, Lines 5-7).

4) How did you determine the sample size? Did you calculate the sample size needed before formal study?

**Response:** Thank you for your question. As noted on Page 11, Line 17, our sample size was based on a study by Bergstrom et al., who used a similar study design.

**Reviewer #5:** This is a generally well written paper. I especially thought the method of generating stereotypes was clever. Thanks for the opportunity to read. Below, a few comments:

1) I don't know that I am convinced by the authors' response to point 3 of the previous review ("Rationale for Selecting Specific Racial Groups"), i.e. by the argument that featuring all groups would limit theoretical precision. This is especially the case because the results are exploratory. In some way, the argument that Arab women are severely underrepresented in research undercuts the point of focusing on "well-documented" stereotypes, especially if most of the participants are majority White. I don't think that all groups need to be represented in this particular study, but the reasoning could be more forthright, whether it be feasibility or something else. Right now, it seems that the manuscript is heavily implying that compared to White women, Asian and Latina women do not have contrasting stereotyped profiles. If this is what the authors believe, it would be helpful to clarify. Regardless, I do think a bit more writing could be done to justify the selection of these groups, and I think the writing could be tightened throughout the paper to make this possible (see points 2-3).

**Response:** Thank you for raising this concern. As noted in the manuscript (Page 11, Line 15), our design was based off a previous study by Bergstrom et al., (2024), who ran a similar study but with male targets. We wanted to extend these findings to women with the same intersecting racial identities used in that study and thus only collected responses pertaining to Black, Arab and White targets. This justification, alongside the justification of how qualitatively different stereotypes are about Black, Arab, and White women (see Page 8-9), is the reason we chose these groups specifically. Furthermore, it would not have been feasible to include more than three groups of comparison, given the complex study design and increased participant sample size this would require.

2) Similarly, it would also be helpful to be a bit more clear about the competing hypotheses you outline in page 8 (perhaps with section headers). I know that the results are exploratory but the results section reads a bit more tightly on the conclusions compared to the introduction. To make this consistent and to not oversell the results, I think you could streamline that front end to say that you are comparing the hypotheses, and also emphasize in the conclusion again the exploratory nature of the results.

**Response:** Thank you for this suggestion. We have adapted the introduction accordingly in order to emphasize the way in which the hypotheses are exploratory in nature (see Page 9, Lines 1-3 and Page 20, Lines 6-8).

3) Consider this statement: "Old Arab women - whose ethnic identity is relatively uncommon and highly salient - were more likely to be stereotyped in line with Arab women (a more distinctive group) than with old women. Old Black women, whose race and age are both socially distinctive, showed a more balanced stereotype." This is an example of a statement that could both be shortened and yet expanded. Specifically, I understood what this meant but it took a few reads to understand -- I would (a) tie this back to the introduction more clearly and the ways you defined socially distinct and (b) shortening the intermediate clauses would tighten this paragraph.

**Response:** Thank you for raising this concern regarding shortening and expanding this section. We have adjusted it accordingly (Page 21, Lines 14-20).

4) The discussion/limitations should also discuss if or how racial stereotypes may have changed in the past few years, as the data was collected prior to 2020.

**Response:** Thank you for this recommendation. I agree that changes in the social and political climate over the last 5 years could influence the results from this study and have noted this in the limitations (Page 24, Lines 13-20).

**Smaller points:**
p. 1 "has demonstrates" -> "has demonstrated"
p. 4 and 5, and throughout the manuscript - the m vs n dash use is not consistent

**Response:** Thank you for catching these oversights. I have adjusted the manuscript accordingly.
