# Supplementary material for: Intersectional stereotypes: How age and race shape perceptions of women in the United States
Source: PLoS One. 2026 Oct 26;21(10):e0361210. doi: 10.1371/journal.pone.0361210 (PMC13704870; doi:10.1371/journal.pone.0361210)
Supplement: Peer review history

# Peer review: Decision Letter 0

**DOI:** 10.1371/journal.pone.0361210.r001

**Authors:** Saeed Ahmad

17 Dec 2025

Dear Dr. Gans,

Thank you for submitting your manuscript to PLOS ONE. After careful consideration, we feel that it has merit but does not fully meet PLOS ONE’s publication criteria as it currently stands. Therefore, we invite you to submit a revised version of the manuscript that addresses the points raised during the review process.

Please submit your revised manuscript by Jan 31 2026 11:59PM. If you will need more time than this to complete your revisions, please reply to this message or contact the journal office at plosone@plos.org. When you're ready to submit your revision, log on to https://www.editorialmanager.com/pone/ and select the 'Submissions Needing Revision' folder to locate your manuscript file.

- A letter that responds to each point raised by the academic editor and reviewer(s). You should upload this letter as a separate file labeled 'Response to Reviewers'.
- A marked-up copy of your manuscript that highlights changes made to the original version. You should upload this as a separate file labeled 'Revised Manuscript with Track Changes'.
- An unmarked version of your revised paper without tracked changes. You should upload this as a separate file labeled 'Manuscript'.

If you would like to make changes to your financial disclosure, please include your updated statement in your cover letter. Guidelines for resubmitting your figure files are available below the reviewer comments at the end of this letter.

If applicable, we recommend that you deposit your laboratory protocols in protocols.io to enhance the reproducibility of your results. Protocols.io assigns your protocol its own identifier (DOI) so that it can be cited independently in the future. For instructions see: https://journals.plos.org/plosone/s/submission-guidelines#loc-laboratory-protocols. Additionally, PLOS ONE offers an option for publishing peer-reviewed Lab Protocol articles, which describe protocols hosted on protocols.io. Read more information on sharing protocols at https://plos.org/protocols?utm_medium=editorial-email&utm_source=authorletters&utm_campaign=protocols.

We look forward to receiving your revised manuscript.

Kind regards,

Saeed Ahmad

Academic Editor

PLOS One

Journal Requirements:

When submitting your revision, we need you to address these additional requirements.

1. Please ensure that your manuscript meets PLOS ONE's style requirements, including those for file naming. The PLOS ONE style templates can be found at

https://journals.plos.org/plosone/s/file?id=wjVg/PLOSOne_formatting_sample_main_body.pdf and

https://journals.plos.org/plosone/s/file?id=ba62/PLOSOne_formatting_sample_title_authors_affiliations.pdf.

2. We note that the grant information you provided in the ‘Funding Information’ and ‘Financial Disclosure’ sections do not match.

When you resubmit, please ensure that you provide the correct grant numbers for the awards you received for your study in the ‘Funding Information’ section.

3. Please be informed that funding information should not appear in the Acknowledgments section or other areas of your manuscript. We will only publish funding information present in the Funding Statement section of the online submission form. Please remove any funding-related text from the manuscript.

4. We note you have included a table to which you do not refer in the text of your manuscript. Please ensure that you refer to Table 2 in your text; if accepted, production will need this reference to link the reader to the Table.

5. Please include a copy of Table 5 which you refer to in your text on page 16.

6. Please include captions for your Supporting Information files at the end of your manuscript, and update any in-text citations to match accordingly. Please see our Supporting Information guidelines for more information: http://journals.plos.org/plosone/s/supporting-information.

7. If the reviewer comments include a recommendation to cite specific previously published works, please review and evaluate these publications to determine whether they are relevant and should be cited. There is no requirement to cite these works unless the editor has indicated otherwise.

[Note: HTML markup is below. Please do not edit.]

Reviewers' comments:

Reviewer's Responses to Questions

**Comments to the Author**

1. Is the manuscript technically sound, and do the data support the conclusions?

Reviewer #1: Partly

Reviewer #2: Yes

**********

2. Has the statistical analysis been performed appropriately and rigorously?

Reviewer #1: I Don't Know

Reviewer #2: Yes

**********

3. Have the authors made all data underlying the findings in their manuscript fully available?

The PLOS Data policy

Reviewer #1: No

Reviewer #2: Yes

**********

4. Is the manuscript presented in an intelligible fashion and written in standard English?

Reviewer #1: No

Reviewer #2: Yes

**********

Reviewer #1: This manuscript examines how intersecting identities of age and race influence stereotypes about women, focusing on older women across three racial groups (Black, White, and Arab). The topic is timely and relevant, given the growing diversity and aging of the U.S. population. The study addresses an important gap by exploring stereotype content at the intersection of age and race for women – a novel contribution, since much past work has focused on either one dimension or on male targets. The manuscript’s strengths include its novelty (investigating understudied combinations of social identities), the thorough data collection and coding of stereotype descriptors from a large sample, and the effort to integrate theoretical frameworks (e.g., intersectionality and distinctiveness principles). These qualities suggest the work has potential to contribute meaningfully to the literature.

However, there are several issues that need to be addressed to strengthen the manuscript. I outline below the major concerns, followed by minor points and general recommendations for improving clarity and tone. My aim is to provide constructive feedback that will help the authors refine their work.

Title Wording (“Shift” Implications): The title “How intersectional identities shift stereotypes for women in the United States…” could be interpreted as implying a causal or dynamic change. The word “shift” suggests that something about having an intersectional identity causes stereotypes to change in a particular direction. However, the study design is cross-sectional and descriptive, comparing stereotype content across groups. There is no temporal shift measured, nor an experimental manipulation to show causation. To avoid misinterpretation, the authors should consider rewording the title to better reflect the content. For example, “How intersectional identities are associated with stereotypes…” or “Intersectional identities and stereotype content: The case of age and race for women in the U.S.”. Such formulations would accurately convey that the study examines differences or associations, rather than implying a causal shift. A clearer title will ensure readers understand the nature of the findings from the outset.

Tone and Claims in the Introduction: Some statements in the Introduction come across as advocacy-driven or overly absolute, which may undermine the scholarly tone. For example, the manuscript states that “Black and Arab people… face some of the most discriminatory treatment at an institutional level…”. This is a strong, broad claim. Even if supported by citations, phrasing it as “the most discriminatory treatment” makes it sound absolute and potentially subjective. I recommend rephrasing such sentences to maintain an objective, evidence-based tone. For instance, the authors could say “Black and Arab people … face very high levels of institutional discrimination (in contexts such as airport security, employment, and law enforcement)”, and ensure each assertion is backed by a citation. This wording conveys the severity of discrimination without implying an unqualified superlative. Overall, the authors should aim to balance the introduction’s passionate framing with neutral scholarly language. This will help the paper come across as objective research rather than advocacy, which in turn can make its conclusions more credible to a scientific audience.

The statement, “To date, most research on people of color has focused on racial groups as a monolith…” overlooks a substantial body of work that has examined intersecting identities beyond race. For instance, numerous studies have explored how factors such as first-generation college student status, socioeconomic class, gender identity, and immigration status intersect with race to shape perceptions and experiences. The authors should revise this claim to more accurately reflect the existing literature and avoid overgeneralization. A more precise framing would strengthen the credibility and scholarly balance of the introduction.

Rationale for Selecting Specific Racial Groups: The choice to focus on Black, White, and Arab women needs a clearer rationale and more consistent presentation. It is valuable that the study goes beyond the Black/White binary; however, the inclusion of Arab women (and not other racial/ethnic groups such as Latina or Asian women) is not fully explained. The Introduction notes that Black and Arab people face high levels of discrimination, but the authors should explicitly clarify why these three groups were selected for analysis over other groups. Is it due to their unique demographic distinctiveness, particular social relevance, or previous literature on these groups? Providing a rationale will help readers understand the scope of the study. Additionally, the manuscript should maintain a consistent order when referring to these groups (sometimes the text reads “Black, White, Arab” and other times “Arab, Black, White”). Choosing one ordering (for example, alphabetical or by population size) and using it throughout will avoid confusion. Clarifying the group selection and ensuring consistency in references will strengthen the study’s focus and coherence. Additionally, if White women are used as a reference group to compare the marginalization of Black and Arab women, the authors should clearly explain why. A brief justification rooted in social hierarchy or prior literature would help strengthen the theoretical framework and avoid confusion.

Inconsistent Ordering of Racial Categories: As noted, the manuscript alternates between listing racial groups in different sequences (“Black, White, Arab” in some places versus “Arab, Black, White” in others). This inconsistency can distract or confuse readers. It would be best to decide on one ordering convention and apply it uniformly throughout the paper. For instance, the authors might choose alphabetical order (Arab, Black, White) or another logical order, but they should stick with it for all mentions of the three groups.

Use of Discipline-Specific Jargon: The text includes quite a bit of specialized terminology (e.g., “intersectional stereotyping,” “diagnosticity principle,” “distinctiveness hypothesis,” “emergent trait content”). While these terms are familiar within social psychology, they may alienate or confuse readers from other backgrounds. To improve accessibility, the authors should define or simplify jargon where possible. For example, when first mentioning the “diagnosticity principle,” provide a brief explanation in plain language (the manuscript does this to some extent by noting it’s about a feature being informative when it distinguishes an individual, which is helpful). Similarly, phrases like “emergent trait content” could be unpacked (e.g., explain that it refers to new stereotype attributes that appear uniquely for the combined identity, rather than being a simple mix of the two single identities’ stereotypes). Ensuring that the prose is understandable to a broad audience will increase the impact of the work, especially in a general-interest journal.

Phrasing and Grammar: There are a few instances of awkward or unclear phrasing and minor grammatical issues. The authors should carefully proofread and possibly have someone not involved in the study read the manuscript to catch confusing sentences. For example, some sentences in the literature review are quite long or double up on ideas, making them hard to follow. Breaking long sentences into two and removing redundant phrases would help. Also, any minor typographical errors or formatting glitches (like inconsistent capitalization, extra spaces, or artifacts from references) should be fixed to polish the presentation. These edits will improve the overall readability and professionalism of the paper.

Reviewer #2: This is a clear and focused study on stereotypes, examining whether stereotypes of older women vary by race (Arab, Black, White) and whether the “most distinctive” identity dimension dominates stereotype content. The design choice – using free-response measures rather than fixed scales – is a particular strength of the study, as it captures culturally available stereotype content without imposing researcher-defined categories.

After a careful review of the data and analyses, I recommend publishing this study. It represents a meaningful contribution, whose strongest claims are transparent, high-quality coding and careful interpretation.

I noted one minor error on page 13: the variable titles in Table 2 appear to be duplicated in the seventh line of the table, likely due to a page break.

**********

what does this mean?). If published, this will include your full peer review and any attached files.

If you choose “no”, your identity will remain anonymous but your review may still be made public.

**Do you want your identity to be public for this peer review?**  For information about this choice, including consent withdrawal, please see our Privacy Policy

Reviewer #1: No

Reviewer #2: No

**********

[NOTE: If reviewer comments were submitted as an attachment file, they will be attached to this email and accessible via the submission site. Please log into your account, locate the manuscript record, and check for the action link "View Attachments". If this link does not appear, there are no attachment files.]

To ensure your figures meet our technical requirements, please review our figure guidelines: https://journals.plos.org/plosone/s/figures

You may also use PLOS’s free figure tool, NAAS, to help you prepare publication quality figures: https://journals.plos.org/plosone/s/figures#loc-tools-for-figure-preparation.

NAAS will assess whether your figures meet our technical requirements by comparing each figure against our figure specifications.

# Author comment: Author response to Decision Letter 1

**DOI:** 10.1371/journal.pone.0361210.r002

15 Jan 2026

This manuscript examines how intersecting identities of age and race influence stereotypes about women, focusing on older women across three racial groups (Black, White, and Arab). The topic is timely and relevant, given the growing diversity and aging of the U.S. population. The study addresses an important gap by exploring stereotype content at the intersection of age and race for women – a novel contribution, since much past work has focused on either one dimension or on male targets. The manuscript’s strengths include its novelty (investigating understudied combinations of social identities), the thorough data collection and coding of stereotype descriptors from a large sample, and the effort to integrate theoretical frameworks (e.g., intersectionality and distinctiveness principles). These qualities suggest the work has potential to contribute meaningfully to the literature.

However, there are several issues that need to be addressed to strengthen the manuscript. I outline below the major concerns, followed by minor points and general recommendations for improving clarity and tone. My aim is to provide constructive feedback that will help the authors refine their work.

1) Title Wording (“Shift” Implications): The title “How intersectional identities shift stereotypes for women in the United States…” could be interpreted as implying a causal or dynamic change. The word “shift” suggests that something about having an intersectional identity causes stereotypes to change in a particular direction. However, the study design is cross-sectional and descriptive, comparing stereotype content across groups. There is no temporal shift measured, nor an experimental manipulation to show causation. To avoid misinterpretation, the authors should consider rewording the title to better reflect the content. For example, “How intersectional identities are associated with stereotypes…” or “Intersectional identities and stereotype content: The case of age and race for women in the U.S.”. Such formulations would accurately convey that the study examines differences or associations, rather than implying a causal shift. A clearer title will ensure readers understand the nature of the findings from the outset.

Response: Thank you for raising this suggestion. We have rewritten the title to better reflect the content.

2) Some statements in the Introduction come across as advocacy-driven or overly absolute, which may undermine the scholarly tone. For example, the manuscript states that “Black and Arab people… face some of the most discriminatory treatment at an institutional level…”. This is a strong, broad claim. Even if supported by citations, phrasing it as “the most discriminatory treatment” makes it sound absolute and potentially subjective. I recommend rephrasing such sentences to maintain an objective, evidence-based tone. For instance, the authors could say “Black and Arab people … face very high levels of institutional discrimination (in contexts such as airport security, employment, and law enforcement)”, and ensure each assertion is backed by a citation. This wording conveys the severity of discrimination without implying an unqualified superlative. Overall, the authors should aim to balance the introduction’s passionate framing with neutral scholarly language. This will help the paper come across as objective research rather than advocacy, which in turn can make its conclusions more credible to a scientific audience.

The statement, “To date, most research on people of color has focused on racial groups as a monolith…” overlooks a substantial body of work that has examined intersecting identities beyond race. For instance, numerous studies have explored how factors such as first-generation college student status, socioeconomic class, gender identity, and immigration status intersect with race to shape perceptions and experiences. The authors should revise this claim to more accurately reflect the existing literature and avoid overgeneralization. A more precise framing would strengthen the credibility and scholarly balance of the introduction.

Response: Thank you for this recommendation. We have refined several areas of the text to reflect more neutral scholarly language rather than advocacy driven statements (see pages 3-4).

3) Rationale for Selecting Specific Racial Groups: The choice to focus on Black, White, and Arab women needs a clearer rationale and more consistent presentation. It is valuable that the study goes beyond the Black/White binary; however, the inclusion of Arab women (and not other racial/ethnic groups such as Latina or Asian women) is not fully explained. The Introduction notes that Black and Arab people face high levels of discrimination, but the authors should explicitly clarify why these three groups were selected for analysis over other groups. Is it due to their unique demographic distinctiveness, particular social relevance, or previous literature on these groups? Providing a rationale will help readers understand the scope of the study. Additionally, the manuscript should maintain a consistent order when referring to these groups (sometimes the text reads “Black, White, Arab” and other times “Arab, Black, White”). Choosing one ordering (for example, alphabetical or by population size) and using it throughout will avoid confusion. Clarifying the group selection and ensuring consistency in references will strengthen the study’s focus and coherence. Additionally, if White women are used as a reference group to compare the marginalization of Black and Arab women, the authors should clearly explain why. A brief justification rooted in social hierarchy or prior literature would help strengthen the theoretical framework and avoid confusion.

Response: Thank you for these suggestions. Because of word count, we are unable to include as many details on the rationale for our choice as we would have liked. However, we have integrated as many relevant details as we could fit (see pages 4-6).

4) Inconsistent Ordering of Racial Categories: As noted, the manuscript alternates between listing racial groups in different sequences (“Black, White, Arab” in some places versus “Arab, Black, White” in others). This inconsistency can distract or confuse readers. It would be best to decide on one ordering convention and apply it uniformly throughout the paper. For instance, the authors might choose alphabetical order (Arab, Black, White) or another logical order, but they should stick with it for all mentions of the three groups.

Response: Thank you for raising these concerns. We have now ordered these groups consistently throughout.

5) Use of Discipline-Specific Jargon: The text includes quite a bit of specialized terminology (e.g., “intersectional stereotyping,” “diagnosticity principle,” “distinctiveness hypothesis,” “emergent trait content”). While these terms are familiar within social psychology, they may alienate or confuse readers from other backgrounds. To improve accessibility, the authors should define or simplify jargon where possible. For example, when first mentioning the “diagnosticity principle,” provide a brief explanation in plain language (the manuscript does this to some extent by noting it’s about a feature being informative when it distinguishes an individual, which is helpful). Similarly, phrases like “emergent trait content” could be unpacked (e.g., explain that it refers to new stereotype attributes that appear uniquely for the combined identity, rather than being a simple mix of the two single identities’ stereotypes). Ensuring that the prose is understandable to a broad audience will increase the impact of the work, especially in a general-interest journal.

Response: Thank you for this suggestion. We have explained the terms respectively throughout (see pages 3 and page 8).

6) Phrasing and Grammar: There are a few instances of awkward or unclear phrasing and minor grammatical issues. The authors should carefully proofread and possibly have someone not involved in the study read the manuscript to catch confusing sentences. For example, some sentences in the literature review are quite long or double up on ideas, making them hard to follow. Breaking long sentences into two and removing redundant phrases would help. Also, any minor typographical errors or formatting glitches (like inconsistent capitalization, extra spaces, or artifacts from references) should be fixed to polish the presentation. These edits will improve the overall readability and professionalism of the paper.

Response: Thank you for drawing our attention to this. We have done our best to edit the manuscript to make it easier to read.

Reviewer #2’s comments

This is a clear and focused study on stereotypes, examining whether stereotypes of older women vary by race (Arab, Black, White) and whether the “most distinctive” identity dimension dominates stereotype content. The design choice – using free-response measures rather than fixed scales – is a particular strength of the study, as it captures culturally available stereotype content without imposing researcher-defined categories.

After a careful review of the data and analyses, I recommend publishing this study. It represents a meaningful contribution, whose strongest claims are transparent, high-quality coding and careful interpretation.

1) I noted one minor error on page 13: the variable titles in Table 2 appear to be duplicated in the seventh line of the table, likely due to a page break.

Response: Thank you for catching this. We have fixed the table accordingly.

Attachment Submitted filename: R&R PlosONE.docx

# Peer review: Decision Letter 1

**DOI:** 10.1371/journal.pone.0361210.r003

**Authors:** Saeed Ahmad

18 May 2026

Dear Dr. Gans,

Thank you for submitting your manuscript to PLOS ONE. After careful consideration, we feel that it has merit but does not fully meet PLOS ONE’s publication criteria as it currently stands. Therefore, we invite you to submit a revised version of the manuscript that addresses the points raised during the review process.

Please submit your revised manuscript by Jul 02 2026 11:59PM. If you will need more time than this to complete your revisions, please reply to this message or contact the journal office at plosone@plos.org. When you're ready to submit your revision, log on to https://www.editorialmanager.com/pone/ and select the 'Submissions Needing Revision' folder to locate your manuscript file.

- A letter that responds to each point raised by the academic editor and reviewer(s). You should upload this letter as a separate file labeled 'Response to Reviewers'.
- A marked-up copy of your manuscript that highlights changes made to the original version. You should upload this as a separate file labeled 'Revised Manuscript with Track Changes'.
- An unmarked version of your revised paper without tracked changes. You should upload this as a separate file labeled 'Manuscript'.

If you would like to make changes to your financial disclosure, please include your updated statement in your cover letter. Guidelines for resubmitting your figure files are available below the reviewer comments at the end of this letter.

If applicable, we recommend that you deposit your laboratory protocols in protocols.io to enhance the reproducibility of your results. Protocols.io assigns your protocol its own identifier (DOI) so that it can be cited independently in the future. For instructions see: https://journals.plos.org/plosone/s/submission-guidelines#loc-laboratory-protocols. Additionally, PLOS ONE offers an option for publishing peer-reviewed Lab Protocol articles, which describe protocols hosted on protocols.io. Read more information on sharing protocols at https://plos.org/protocols?utm_medium=editorial-email&utm_source=authorletters&utm_campaign=protocols.

As the corresponding author, your ORCID iD is verified in the submission system and will appear in the published article. PLOS supports the use of ORCID, and we encourage all coauthors to register for an ORCID iD and use it as well. Please encourage your coauthors to verify their ORCID iD within the submission system before final acceptance, as unverified ORCID iDs will not appear in the published article. *Only*  the individual author can complete the verification step; PLOS staff *cannot*  verify ORCID iDs on behalf of authors.

We look forward to receiving your revised manuscript.

Kind regards,

Saeed Ahmad

Academic Editor

PLOS One

Journal Requirements:

If the reviewer comments include a recommendation to cite specific previously published works, please review and evaluate these publications to determine whether they are relevant and should be cited. There is no requirement to cite these works unless the editor has indicated otherwise.

Please review your reference list to ensure that it is complete and correct. If you have cited papers that have been retracted, please include the rationale for doing so in the manuscript text, or remove these references and replace them with relevant current references. Any changes to the reference list should be mentioned in the rebuttal letter that accompanies your revised manuscript. If you need to cite a retracted article, indicate the article’s retracted status in the References list and also include a citation and full reference for the retraction notice.

[Note: HTML markup is below. Please do not edit.]

Reviewers' comments:

Reviewer's Responses to Questions

**Comments to the Author**

Reviewer #3: All comments have been addressed

Reviewer #4: (No Response)

Reviewer #5: (No Response)

**********

2. Is the manuscript technically sound, and do the data support the conclusions?

Reviewer #3: Yes

Reviewer #4: (No Response)

Reviewer #5: Partly

**********

3. Has the statistical analysis been performed appropriately and rigorously?

Reviewer #3: Yes

Reviewer #4: (No Response)

Reviewer #5: Yes

**********

4. Have the authors made all data underlying the findings in their manuscript fully available?

The PLOS Data policy

Reviewer #3: Yes

Reviewer #4: (No Response)

Reviewer #5: Yes

**********

5. Is the manuscript presented in an intelligible fashion and written in standard English?

Reviewer #3: Yes

Reviewer #4: (No Response)

Reviewer #5: Yes

**********

Reviewer #3: The authors should be commended for their thoughtful work moving the literature forward. The authors have clearly addressed prior reviewer comments. Below are suggestions to strengthen the manuscript, especially regarding intersectionality and presentation of findings.

Major suggestion:

Intersectionality: The manuscript currently treats intersectionality mainly as an analytic framework for intersecting individual identities. Expand this to emphasize that intersectionality links intersecting identities (e.g., race, gender, age) to social structures and processes (e.g., racism, sexism, ageism) that produce unequal outcomes. This theoretical expansion should be reflected in the definition, analytic approach, interpretation of results, and implications. Useful references to incorporate:

Agénor, M. (2020). Future directions for incorporating intersectionality into quantitative population health research. American Journal of Public Health, 110(6), 803–806.

Aguayo-Romero, R. A. (2021). (Re)centering Black feminism into intersectionality research. American Journal of Public Health, 111(1), 101–103.

Moradi, B., Parent, M. C., Weis, A. S., Ouch, S., & Broad, K. L. (2020). Mapping the travels of intersectionality scholarship: A citation network analysis. Psychology of Women Quarterly, 44(2), 151–169.

Interpretation: Where the results are discussed as supporting a “distinctiveness hypothesis” rather than an “intersectional hypothesis,” clarify what this means substantively. Explain implications for understanding how group-specific stereotypes or experiences operate, and how structural processes (rather than only individual identity combinations) might explain these patterns.

Specific edits / minor comments:

Pg. 6, line 77: Expand the definition of intersectionality to state explicitly that it is not only about intersecting individual identities but about how those identities are linked to and produced by social structures and power relations (e.g., racism, sexism, classism).

Pg. 17, line 321: Check count — there are six stereotypes listed for older White women, not five.

Pg. 20, line 370: There are six stereotypes listed for Black women — please verify and correct count/description.

Pg. 22, line 400: There are six stereotypes listed for Arab women and older Arab women — please verify and correct.

Pg. 27, line 488: Clarify the meaning and implications of finding more evidence for a distinctiveness hypothesis than an intersectional hypothesis. What does this imply about mechanisms, policy, or intervention priorities?

Implications and recommendations:

Expand the implications section. Specifically spell out what the findings mean for Black and Arab women in practical terms (e.g., experiences of discrimination, health, social opportunities).

Discuss how these results could inform tailored interventions or policies (e.g., stereotype reduction campaigns, culturally specific support programs, structural interventions to address racism/sexism).

If possible, suggest concrete next steps for research that would test structural mechanisms (e.g., measures of exposure to racism or sexism, policy variation, multilevel models).

Overall assessment, this is a strong and important manuscript with careful revisions already completed. Strengthening the conceptualization and application of intersectionality and expanding the implications and interpretation will substantially improve the paper’s contribution.

Reviewer #4: “Intersectional Stereotypes: How Age and Race Shape Perceptions of Women in the United States”（PONE-D-25-56215R1）

Thanks for the invitation. I am not the original reviewer of this manuscript. This investigation aimed to examine the influence of older age and race (White/Black/Arab) on stereotypes of women. The study found stronger support for a distinctiveness hypothesis, whereby the most distinct identity of an individual's intersectional identity influenced their associated characteristics. Overall, this topic is interesting and the findings hold some theoretical and practical implications. However, some concerns appeared after reading the whole manuscript.

1. Some important papers need to be reviewed and discussed, such as,

Hudson, S. K. T. J., Myer, A., & Berney, E. C. (2024). Stereotyping, prejudice, and discrimination at the intersection of race and gender: An intersectional theory primer. Social and Personality Psychology Compass, 18(2), e12939.

Petsko, C. D., Rosette, A. S., & Bodenhausen, G. V. (2022). Through the looking glass: A lens-based account of intersectional stereotyping. Journal of personality and social psychology, 123(4), 763.

Rosenthal, L., Overstreet, N. M., Khukhlovich, A., Brown, B. E., Godfrey, C. J., & Albritton, T. (2020). Content of, sources of, and responses to sexual stereotypes of Black and Latinx women and men in the United States: A qualitative intersectional exploration. Journal of Social Issues, 76(4), 921-948.

Hand, M. D., & Ihara, E. S. (2024). Ageism, racism, sexism, and work with older healthcare clients: Why an intersectional approach is needed in practice, policy, education, and research. The International Journal of Aging and Human Development, 98(1), 27-38.

2. The theoretical implication can be further discussed with the follow framework.

Smith-Tran, A. (2025). Age-Centered Racism: An Emergent Framework for Considering How Ageism and Racism Intersect. Sociological Research Online, 13607804251382021.

3. For the stereotype content of White, Black, Arab Women Stereotypes, I believe the findings from the Stereotype Content Model would provide much more directly-related evidence and should be reviewed and discussed in the current manuscript. Please do not worry about the word limit since the Plos one is an open access journal without specific page limit.

4. How did you determine the sample size? Did you calculate the sample size needed before formal study?

Reviewer #5: This is a generally well written paper. I especially thought the method of generating stereotypes was clever. Thanks for the opportunity to read. Below, a few comments:

1. I don't know that I am convinced by the authors' response to point 3 of the previous review ("Rationale for Selecting Specific Racial Groups"), i.e. by the argument that featuring all groups would limit theoretical precision. This is especially the case because the results are exploratory. In some way, the argument that Arab women are severely underrepresented in research undercuts the point of focusing on "well-documented" stereotypes, especially if most of the participants are majority White.

I don't think that all groups need to be represented in this paritcular study, but the reasoning could be more forthright, whether it be feasibility or something else. Right now, it seems that the manuscript is heavily implying that compared to White women, Asian and Latina women do not have contrasting stereotyped profiles. If this is what the authors believe, it would be helpful to clarify. Regardless, I do think a bit more writing could be done to justify the selection of these groups, and I think the writing could be tightened throughout the paper to make this possible (see points 2-3).

2. Similarly, it would also be helpful to be a bit more clear about the competing hypotheses you outline in page 8 (perhaps with section headers). I know that the results are exploratory but the results section reads a bit more tightly on the conclusions compared to the introduction. To make this consistent and to not oversell the results, I think you could streamline that front end to say that you are comparing the hypotheses, and also emphasize in the conclusion again the exploratory nature of the reuslts.

3. Consider this statement: "Old Arab women - whose ethnic identity is relatively uncommon and highly salient - were more likely to be stereotyped in line with Arab women (a more distinctive group) than with old women. Old Black women, whose race and age are both socially distinctive, showed a more balanced stereotype." This is an example of a statement that could both be shortened and yet expanded. Specifically, I understood what this meant but it took a few reads to understand -- I would (a) tie this back to the introduction more clearly and the ways you defined socially distinct and (b) shortening the intermediate clauses would tighten this paragraph.

4. The discussion/limitations should also discuss if or how racial stereotypes may have changed in the past few years, as the data was collected prior to 2020.

Smaller points:

p. 1 "has demonstrates" -> "has demonstrated"

p. 4 and 5, and throughout the manuscript - the m vs n dash use is not consistent

**********

what does this mean?). If published, this will include your full peer review and any attached files.

If you choose “no”, your identity will remain anonymous but your review may still be made public.

**Do you want your identity to be public for this peer review?**  For information about this choice, including consent withdrawal, please see our Privacy Policy

Reviewer #3: No

Reviewer #4: No

Reviewer #5: No

**********

[NOTE: If reviewer comments were submitted as an attachment file, they will be attached to this email and accessible via the submission site. Please log into your account, locate the manuscript record, and check for the action link "View Attachments". If this link does not appear, there are no attachment files.]

To ensure your figures meet our technical requirements, please review our figure guidelines: https://journals.plos.org/plosone/s/figures

You may also use PLOS’s free figure tool, NAAS, to help you prepare publication quality figures: https://journals.plos.org/plosone/s/figures#loc-tools-for-figure-preparation.

NAAS will assess whether your figures meet our technical requirements by comparing each figure against our figure specifications.

# Author comment: Author response to Decision Letter 2

**DOI:** 10.1371/journal.pone.0361210.r004

22 Jun 2026

22-June-2026

Dr. Emily Chenette

Editor in-Chief, PLOS One

Dear Dr. Chenette,

Thank you for the thoughtful review of the first revision our manuscript PONE-D-25-56215 entitled " Intersectional Stereotypes: How Age and Race Shape Perceptions of Women in the United States". We greatly appreciate that the reviewers noted that our revised manuscript continues to address an important topic, and that the paper has the potential to provide a meaningful contribution by providing insight into how race and age intersect with gender to impact person perception.

Below, we address each point made by you and the reviewers. We consider this second revision of the paper, which we have revised in accordance with the suggestions provided, to have strengthened the manuscript. We hope that our revisions address your concerns and that you will now find the paper to be suitable for publication.

Best regards,

Hannah Gans, Alison L. Chasteen.

Reviewer #3: The authors should be commended for their thoughtful work moving the literature forward. The authors have clearly addressed prior reviewer comments. Below are suggestions to strengthen the manuscript, especially regarding intersectionality and presentation of findings.

We thank the reviewer for their positive comments regarding the potential contribution of this work and of our efforts at addressing reviewer suggestions.

Major suggestion:

1) Intersectionality: The manuscript currently treats intersectionality mainly as an analytic framework for intersecting individual identities. Expand this to emphasize that intersectionality links intersecting identities (e.g., race, gender, age) to social structures and processes (e.g., racism, sexism, ageism) that produce unequal outcomes. This theoretical expansion should be reflected in the definition, analytic approach, interpretation of results, and implications. Useful references to incorporate:

Agénor, M. (2020). Future directions for incorporating intersectionality into quantitative population health research. American Journal of Public Health, 110(6), 803–806.

Aguayo-Romero, R. A. (2021). (Re)centering Black feminism into intersectionality research. American Journal of Public Health, 111(1), 101–103.

Moradi, B., Parent, M. C., Weis, A. S., Ouch, S., & Broad, K. L. (2020). Mapping the travels of intersectionality scholarship: A citation network analysis. Psychology of Women Quarterly, 44(2), 151–169.

Response: Thank you for these suggestions. We have expanded the way intersectionality is described to reflect the fact that it links intersecting identities (e.g., race, gender, age) to social structures and processes (e.g., racism, sexism, ageism) that produce unequal outcomes, and have incorporated the recommended references in these areas (see page 3, lines 20-23- page 4, lines 1-3; page 9, lines 4-12; page 20, lines 10-13).

2)Interpretation: Where the results are discussed as supporting a “distinctiveness hypothesis” rather than an “intersectional hypothesis,” clarify what this means substantively. Explain implications for understanding how group-specific stereotypes or experiences operate, and how structural processes (rather than only individual identity combinations) might explain these patterns.

Response: Thank you for this recommendation. We have described the implications for our results in further detail (page 22, lines 17-23; page 23, lines 1-23).

Specific edits / minor comments:

1) Pg. 6, line 77: Expand the definition of intersectionality to state explicitly that it is not only about intersecting individual identities but about how those identities are linked to and produced by social structures and power relations (e.g., racism, sexism, classism).

Response: Thank you for this suggestion. We have expanded the way intersectionality is described to reflect the fact that it links intersecting identities (e.g., race, gender, age) to social structures and processes (e.g., racism, sexism, ageism) that produce unequal outcomes.

2) Pg. 17, line 321: Check count — there are six stereotypes listed for older White women, not five.

Pg. 20, line 370: There are six stereotypes listed for Black women — please verify and correct count/description. Pg. 22, line 400: There are six stereotypes listed for Arab women and older Arab women — please verify and correct.

Response: Thank you for catching this oversight. We have verified and corrected these numbers.

3) Pg. 27, line 488: Clarify the meaning and implications of finding more evidence for a distinctiveness hypothesis than an intersectional hypothesis. What does this imply about mechanisms, policy, or intervention priorities?

Response: Thank you for this suggestion. We have clarified the meaning and implications for finding mor evidence for a distinctiveness hypothesis compared to an intersectional hypothesis (page 22, lines 17-23; page 23, lines 1-23).

Implications and recommendations:

1) Expand the implications section. Specifically spell out what the findings mean for Black and Arab women in practical terms (e.g., experiences of discrimination, health, social opportunities).

Response: Thank you for this suggestion. We have expanded on what the findings mean for Black and Arab women in practical terms (see Page 23, Lines 1-7).

2) Discuss how these results could inform tailored interventions or policies (e.g., stereotype reduction campaigns, culturally specific support programs, structural interventions to address racism/sexism). If possible, suggest concrete next steps for research that would test structural mechanisms (e.g., measures of exposure to racism or sexism, policy variation, multilevel models).

Response: Thank you for this suggestion. We have expanded on how these results could inform tailored interventions or policies, as well as concrete next steps for research (see Page 23, Lines 8-23)

Reviewer #4: Overall assessment, this is a strong and important manuscript with careful revisions already completed. Strengthening the conceptualization and application of and expanding the implications and interpretation will substantially improve the paper’s contribution.

Thanks for the invitation. I am not the original reviewer of this manuscript. This investigation aimed to examine the influence of older age and race (White/Black/Arab) on stereotypes of women. The study found stronger support for a distinctiveness hypothesis, whereby the most distinct identity of an individual's intersectional identity influenced their associated characteristics. Overall, this topic is interesting, and the findings hold some theoretical and practical implications. However, some concerns appeared after reading the whole manuscript.

Response: We are gratified that the Reviewer feels this is a strong and important manuscript and that the topic is interesting and that the findings hold theoretical and practical implications. We also appreciate the acknowledgement that we had already completed careful revisions and the suggestions for further strengthening of the paper.

1) Some important papers need to be reviewed and discussed, such as,

Hudson, S. K. T. J., Myer, A., & Berney, E. C. (2024). Stereotyping, prejudice, and discrimination at the intersection of race and gender: An intersectional theory primer. Social and Personality Psychology Compass, 18(2), e12939.

Petsko, C. D., Rosette, A. S., & Bodenhausen, G. V. (2022). Through the looking glass: A lens-based account of intersectional stereotyping. Journal of personality and social psychology, 123(4), 763.

Rosenthal, L., Overstreet, N. M., Khukhlovich, A., Brown, B. E., Godfrey, C. J., & Albritton, T. (2020). Content of, sources of, and responses to sexual stereotypes of Black and Latinx women and men in the United States: A qualitative intersectional exploration. Journal of Social Issues, 76(4), 921-948.

Hand, M. D., & Ihara, E. S. (2024). Ageism, racism, sexism, and work with older healthcare clients: Why an intersectional approach is needed in practice, policy, education, and research. The International Journal of Aging and Human Development, 98(1), 27-38.

Response: Thank you for this suggestion. We have included these references throughout.

2) The theoretical implication can be further discussed with the follow framework.

Smith-Tran, A. (2025). Age-Centered Racism: An Emergent Framework for Considering How Ageism and Racism Intersect. Sociological Research Online, 13607804251382021.

Response: Thank you for this recommendation. We have expanded the theoretical implications with the use of these references (Page 22, lines 17-23; Page 23, lines 1-23).

3) For the stereotype content of White, Black, Arab Women Stereotypes, I believe the findings from the Stereotype Content Model would provide much more directly-related evidence and should be reviewed and discussed in the current manuscript. Please do not worry about the word limit since the Plos one is an open access journal without specific page limit.

Response: Thank you for this recommendation. We have included a description of the Stereotype Content Model as well as predictions based off of this model into the manuscript (see Page 4, Lines 9-14, Lines 17-19; Page 5, Lines 5-7).

4) How did you determine the sample size? Did you calculate the sample size needed before formal study?

Response: Thank you for your question. As noted on Page 11, Line 17, our sample size was based on a study by Bergstrom et al., who used a similar study design.

Reviewer #5: This is a generally well written paper. I especially thought the method of generating stereotypes was clever. Thanks for the opportunity to read. Below, a few comments:

1) I don't know that I am convinced by the authors' response to point 3 of the previous review ("Rationale for Selecting Specific Racial Groups"), i.e. by the argument that featuring all groups would limit theoretical precision. This is especially the case because the results are exploratory. In some way, the argument that Arab women are severely underrepresented in research undercuts the point of focusing on "well-documented" stereotypes, especially if most of the participants are majority White. I don't think that all groups need to be represented in this particular study, but the reasoning could be more forthright, whether it be feasibility or something else. Right now, it seems that the manuscript is heavily implying that compared to White women, Asian and Latina women do not have contrasting stereotyped profiles. If this is what the authors believe, it would be helpful to clarify. Regardless, I do think a bit more writing could be done to justify the selection of these groups, and I think the writing could be tightened throughout the paper to make this possible (see points 2-3).

Response: Thank you for raising this concern. As noted in the manuscript (Page 11, Line 15), our design was based off a previous study by Bergstrom et al., (2024), who ran a similar study but with male targets. We wanted to extend these findings to women with the same intersecting racial identities used in that study and thus only collected responses pertaining to Black, Arab and White targets. This justification, alongside the justification of how qualitatively different stereotypes are about Black, Arab, and White women (see Page 8-9), is the reason we chose these groups specifically. Furthermore, it would not have been feasible to include more than three groups of comparison, given the complex study design and increased participant sample size this would require.

2) Similarly, it would also be helpful to be a bit more clear about the competing hypotheses you outline in page 8 (perhaps with section headers). I know that the results are exploratory but the results section reads a bit more tightly on the conclusions compared to the introduction. To make this consistent and to not oversell the results, I think you could streamline that front end to say that you are comparing the hypotheses, and also emphasize in the conclusion again the exploratory nature of the results.

Response: Thank you for this suggestion. We have adapted the introduction accordingly in order to emphasize the way in which the hypotheses are exploratory in nature (see Page 9, Lines 1-3 and Page 20, Lines 6-8).

3) Consider this statement: "Old Arab women - whose ethnic identity is relatively uncommon and highly salient - were more likely to be stereotyped in line with Arab women (a more distinctive group) than with old women. Old Black women, whose race and age are both socially distinctive, showed a more balanced stereotype." This is an example of a statement that could both be shortened and yet expanded. Specifically, I understood what this meant but it took a few reads to understand -- I would (a) tie this back to the introduction more clearly and the ways you defined socially distinct and (b) shortening the intermediate clauses would tighten this paragraph.

Response: Thank you for raising this concern regarding shortening and expanding this section. We have adjusted it accordingly (Page 21, Lines 14-20).

4) The discussion/limitations should also discuss if or how racial stereotypes may have changed in the past few years, as the data was collected prior to 2020.

Response: Thank you for this recommendation. I agree that changes in the social and political climate over the last 5 years could influence the results from this study and have noted this in the limitations (Page 24, Lines 13-20).

Smaller points:

p. 1 "has demonstrates" -> "has demonstrated"

p. 4 and 5, and throughout the manuscript - the m vs n dash use is not consistent

Response: Thank you for catching these oversights. I have adjusted the manuscript accordingly.

Attachment Submitted filename: Response to PLOS one reviews.docx

# Peer review: Decision Letter 2

**DOI:** 10.1371/journal.pone.0361210.r005

**Authors:** Saeed Ahmad

6 Jul 2026

Dear Dr. Gans,

Thank you for submitting your manuscript to PLOS ONE. After careful consideration, we feel that it has merit but does not fully meet PLOS ONE’s publication criteria as it currently stands. Therefore, we invite you to submit a revised version of the manuscript that addresses the points raised during the review process.

Please submit your revised manuscript by Aug 20 2026 11:59PM. If you will need more time than this to complete your revisions, please reply to this message or contact the journal office at plosone@plos.org. When you're ready to submit your revision, log on to https://www.editorialmanager.com/pone/ and select the 'Submissions Needing Revision' folder to locate your manuscript file.

- A letter that responds to each point raised by the academic editor and reviewer(s). You should upload this letter as a separate file labeled 'Response to Reviewers'.
- A marked-up copy of your manuscript that highlights changes made to the original version. You should upload this as a separate file labeled 'Revised Manuscript with Track Changes'.
- An unmarked version of your revised paper without tracked changes. You should upload this as a separate file labeled 'Manuscript'.

If you would like to make changes to your financial disclosure, please include your updated statement in your cover letter. Guidelines for resubmitting your figure files are available below the reviewer comments at the end of this letter.

If applicable, we recommend that you deposit your laboratory protocols in protocols.io to enhance the reproducibility of your results. Protocols.io assigns your protocol its own identifier (DOI) so that it can be cited independently in the future. For instructions see: https://journals.plos.org/plosone/s/submission-guidelines#loc-laboratory-protocols. Additionally, PLOS ONE offers an option for publishing peer-reviewed Lab Protocol articles, which describe protocols hosted on protocols.io. Read more information on sharing protocols at https://plos.org/protocols?utm_medium=editorial-email&utm_source=authorletters&utm_campaign=protocols.

As the corresponding author, your ORCID iD is verified in the submission system and will appear in the published article. PLOS supports the use of ORCID, and we encourage all coauthors to register for an ORCID iD and use it as well. Please encourage your coauthors to verify their ORCID iD within the submission system before final acceptance, as unverified ORCID iDs will not appear in the published article. *Only*  the individual author can complete the verification step; PLOS staff *cannot*  verify ORCID iDs on behalf of authors.

We look forward to receiving your revised manuscript.

Kind regards,

Saeed Ahmad, PhD

Academic Editor

PLOS One

Journal Requirements:

1. If the reviewer comments include a recommendation to cite specific previously published works, please review and evaluate these publications to determine whether they are relevant and should be cited. There is no requirement to cite these works unless the editor has indicated otherwise.

2. Please review your reference list to ensure that it is complete and correct. If you have cited papers that have been retracted, please include the rationale for doing so in the manuscript text, or remove these references and replace them with relevant current references. Any changes to the reference list should be mentioned in the rebuttal letter that accompanies your revised manuscript. If you need to cite a retracted article, indicate the article’s retracted status in the References list and also include a citation and full reference for the retraction notice.

[Note: HTML markup is below. Please do not edit.]

Reviewers' comments:

Reviewer's Responses to Questions

**Comments to the Author**

Reviewer #4: (No Response)

Reviewer #5: (No Response)

**********

2. Is the manuscript technically sound, and do the data support the conclusions?

Reviewer #4: Yes

Reviewer #5: Partly

**********

3. Has the statistical analysis been performed appropriately and rigorously?

Reviewer #4: Yes

Reviewer #5: I Don't Know

**********

4. Have the authors made all data underlying the findings in their manuscript fully available?

The PLOS Data policy

Reviewer #4: Yes

Reviewer #5: Yes

**********

5. Is the manuscript presented in an intelligible fashion and written in standard English?

Reviewer #4: Yes

Reviewer #5: Yes

**********

Reviewer #4: Thanks for the revisions and one concern remains. The following article which was mentioned in my previous round review and is relevant and should be reviewed and discussed in the current manuscript.

Rosenthal, L., Overstreet, N. M., Khukhlovich, A., Brown, B. E., Godfrey, C. J., &

Albritton, T. (2020). Content of, sources of, and responses to sexual stereotypes of

Black and Latinx women and men in the United States: A qualitative intersectional

exploration. Journal of Social Issues, 76(4), 921-948.

Reviewer #5: Thank you for your revision. Most of my comments have been addressed, and the additional justification you have provided on page 8-9 regarding why Black and Arab female stereotypes was especially helpful.

However, in light of that, I do think that it would be worthwhile to remove the sentence on page 8, lines 5-7: "The present approach prioritizes depth over breadth by focusing on groups with well-documented but contrasting stereotype profiles." What I wrote in my original review was that this particular phrase essentially states that Asian and Latina women DO NOT have "well-documented but contrasting stereotype profiles."

To be clear, I think your justification on pages 8-9 is sufficient, and I do not disagree with you on sample feasibility. But not only do I find this statement to be empirically untrue, it risks reinforcing stereotypes about Asian/Latina women - especially hard to reconcile with the subject of this paper. Given your excellent explanation afterward, I'm unsure why it was left in.

**********

what does this mean?). If published, this will include your full peer review and any attached files.

If you choose “no”, your identity will remain anonymous but your review may still be made public.

**Do you want your identity to be public for this peer review?**  For information about this choice, including consent withdrawal, please see our Privacy Policy

Reviewer #4: No

Reviewer #5: No

**********

[NOTE: If reviewer comments were submitted as an attachment file, they will be attached to this email and accessible via the submission site. Please log into your account, locate the manuscript record, and check for the action link "View Attachments". If this link does not appear, there are no attachment files.]

To ensure your figures meet our technical requirements, please review our figure guidelines: https://journals.plos.org/plosone/s/figures

You may also use PLOS’s free figure tool, NAAS, to help you prepare publication quality figures: https://journals.plos.org/plosone/s/figures#loc-tools-for-figure-preparation.

NAAS will assess whether your figures meet our technical requirements by comparing each figure against our figure specifications.

# Author comment: Author response to Decision Letter 3

**DOI:** 10.1371/journal.pone.0361210.r006

4 Sep 2026

07-July-2026

Dr. Emily Chenette

Editor in-Chief, PLOS One

Dear Dr. Chenette,

Thank you for the thoughtful review of the second revision our manuscript PONE-D-25-56215 entitled " Intersectional Stereotypes: How Age and Race Shape Perceptions of Women in the United States". We greatly appreciate that the reviewers noted that our revised manuscript continues to address an important topic, and that the paper has the potential to provide a meaningful contribution by providing insight into how race and age intersect with gender to impact person perception.

Below, we address the final points made by the reviewers. We consider this third revision of the paper, which we have revised in accordance with the suggestions provided, to have strengthened the manuscript. We hope that our revisions address your concerns and that you will now find the paper to be suitable for publication.

Best regards,

Hannah Gans, Alison L. Chasteen.

Reviewer #4: Thanks for the revisions and one concern remains. The following article which was mentioned in my previous round review and is relevant and should be reviewed and discussed in the current manuscript.

Rosenthal, L., Overstreet, N. M., Khukhlovich, A., Brown, B. E., Godfrey, C. J., &

Albritton, T. (2020). Content of, sources of, and responses to sexual stereotypes of

Black and Latinx women and men in the United States: A qualitative intersectional

exploration. Journal of Social Issues, 76(4), 921-948.

Response: Thank you for catching this oversight. We have reviewed this article and have integrated it into the manuscript on page 9 line 1.

Reviewer #5: Thank you for your revision. Most of my comments have been addressed, and the additional justification you have provided on page 8-9 regarding why Black and Arab female stereotypes was especially helpful.

However, in light of that, I do think that it would be worthwhile to remove the sentence on page 8, lines 5-7: "The present approach prioritizes depth over breadth by focusing on groups with well-documented but contrasting stereotype profiles." What I wrote in my original review was that this particular phrase essentially states that Asian and Latina women DO NOT have "well-documented but contrasting stereotype profiles."

To be clear, I think your justification on pages 8-9 is sufficient, and I do not disagree with you on sample feasibility. But not only do I find this statement to be empirically untrue, it risks reinforcing stereotypes about Asian/Latina women - especially hard to reconcile with the subject of this paper. Given your excellent explanation afterward, I'm unsure why it was left in.

Response: Thank you for this recommendation. We agree with this and have removed the sentence on page 8 lines 5-7.

Attachment Submitted filename: Response to PLOSONE reviews.docx

# Peer review: Decision Letter 3

**DOI:** 10.1371/journal.pone.0361210.r007

**Authors:** Saeed Ahmad

7 Oct 2026

Intersectional Stereotypes: How Age and Race Shape Perceptions of Women in the United States

PONE-D-25-56215R3

Dear Dr. Gans,

We’re pleased to inform you that your manuscript has been judged scientifically suitable for publication and will be formally accepted for publication once it meets all outstanding technical requirements.

Within one week, you’ll receive an e-mail detailing the required amendments. When these have been addressed, you’ll receive a formal acceptance letter and your manuscript will be scheduled for publication.

An invoice will be generated when your article is formally accepted. Please note, if your institution has a publishing partnership with PLOS and your article meets the relevant criteria, all or part of your publication costs will be covered. Please make sure your user information is up-to-date by logging into Editorial Manager at Editorial Manager® and clicking the ‘Update My Information' link at the top of the page. For questions related to billing, please contact billing support.

If your institution or institutions have a press office, please notify them about your upcoming paper to help maximize its impact. If they’ll be preparing press materials, please inform our press team as soon as possible -- no later than 48 hours after receiving the formal acceptance. Your manuscript will remain under strict press embargo until 2 pm Eastern Time on the date of publication. For more information, please contact onepress@plos.org.

Kind regards,

Saeed Ahmad

Academic Editor

PLOS One

Additional Editor Comments (optional):

Reviewers' comments:

Reviewer's Responses to Questions

**Comments to the Author**

Reviewer #4: (No Response)

**********

2. Is the manuscript technically sound, and do the data support the conclusions?

Reviewer #4: (No Response)

**********

3. Has the statistical analysis been performed appropriately and rigorously?

Reviewer #4: (No Response)

**********

4. Have the authors made all data underlying the findings in their manuscript fully available?

The PLOS Data policy

Reviewer #4: (No Response)

**********

5. Is the manuscript presented in an intelligible fashion and written in standard English?

Reviewer #4: (No Response)

**********

Reviewer #4: (No Response)

**********

what does this mean?). If published, this will include your full peer review and any attached files.

If you choose “no”, your identity will remain anonymous but your review may still be made public.

**Do you want your identity to be public for this peer review?**  For information about this choice, including consent withdrawal, please see our Privacy Policy

Reviewer #4: No

**********

# Editor report: Acceptance letter

**DOI:** 10.1371/journal.pone.0361210.r008

**Authors:** Saeed Ahmad

PONE-D-25-56215R3

PLOS One

Dear Dr. Gans,

I'm pleased to inform you that your manuscript has been deemed suitable for publication in PLOS One. Congratulations! Your manuscript is now being handed over to our production team.

At this stage, our production department will prepare your paper for publication. This includes ensuring the following:

* All references, tables, and figures are properly cited

* All relevant supporting information is included in the manuscript submission,

* There are no issues that prevent the paper from being properly typeset

You will receive further instructions from the production team, including instructions on how to review your proof when it is ready. Please keep in mind that we are working through a large volume of accepted articles, so please give us a few days to review your paper and let you know the next and final steps.

Lastly, if your institution or institutions have a press office, please let them know about your upcoming paper now to help maximize its impact. If they'll be preparing press materials, please inform our press team within the next 48 hours. Your manuscript will remain under strict press embargo until 2 pm Eastern Time on the date of publication. For more information, please contact onepress@plos.org.

You will receive an invoice from PLOS for your publication fee after your manuscript has reached the completed accept phase. If you receive an email requesting payment before acceptance or for any other service, this may be a phishing scheme. Learn how to identify phishing emails and protect your accounts at https://explore.plos.org/phishing.

If we can help with anything else, please email us at customercare@plos.org.

Thank you for submitting your work to PLOS One and supporting open access.

Kind regards,

PLOS One Editorial Office Staff

on behalf of

Dr. Saeed Ahmad

Academic Editor

PLOS One
